# The RACK1 signal anchor protein from *Trypanosoma brucei* associates with eukaryotic elongation factor 1A: a role for translational control in cytokinesis

**DOI:** 10.1111/j.1365-2958.2008.06443.x

**Published:** 2008-09-25

**Authors:** Sandesh Regmi, Karen G Rothberg, James G Hubbard, Larry Ruben

**Affiliations:** Department of Biological Sciences, Southern Methodist UniversityDallas, TX 75275, USA

## Abstract

RACK1 is a WD-repeat protein that forms signal complexes at appropriate locations in the cell. RACK1 homologues are core components of ribosomes from yeast, plants and mammals. In contrast, a cryo-EM analysis of trypanosome ribosomes failed to detect RACK1, thus eliminating an important translational regulatory mechanism. Here we report that TbRACK1 from *Trypanosoma brucei* associates with eukaryotic translation elongation factor-1a (eEF1A) as determined by tandem MS of TAP-TbRACK1 affinity eluates, co-sedimentation in a sucrose gradient, and co-precipitation assays. Consistent with these observations, sucrose gradient purified 80S monosomes and translating polysomes each contained TbRACK1. When RNAi was used to deplete cells of TbRACK1, a shift in the polysome profile was observed, while the phosphorylation of a ribosomal protein increased. Under these conditions, cell growth became hypersensitive to the translational inhibitor anisomycin. The kinetoplasts and nuclei were misaligned in the postmitotic cells, resulting in partial cleavage furrow ingression during cytokinesis. Overall, these findings identify eEF1A as a novel TbRACK1 binding partner and establish TbRACK1 as a component of the trypanosome translational apparatus. The synergy between anisomycin and TbRACK1 RNAi suggests that continued translation is required for complete ingression of the cleavage furrow.

## Introduction

Selective changes in protein composition accompany both the cell cycle and complex life cycle of *Trypanosoma brucei*. The mechanisms that account for these changes are only partially understood (Reviewed in Clayton, 2002; Campbell *et al*., 2003; Clayton and Shapira, 2007). Because trypanosome genes are organized as long cotranscribed polycistronic units, transcriptional control is not likely to play a major role in differential gene expression. Trypanosomes therefore rely on a combination of post-transcriptional control, translational control and proteolysis to alter steady-state levels of proteins in the cell. Considerable attention has been directed towards regulatory events that affect mRNA abundance. In this regard, mRNA processing rates and mRNA stability are important (Clayton and Shapira, 2007). A growing number of studies also demonstrate a change in protein abundance with minimal change in mRNA levels. This situation has been demonstrated for individual proteins and by genome-wide analyses that compare transciptome and proteome data sets (Gale *et al*., 1994; Saas *et al*., 2000; Avila *et al*., 2001; Akopyants *et al*., 2004; Brems *et al*., 2005; McNicoll *et al*., 2006; Mayho *et al*., 2006; Cohen-Freue *et al*., 2007; Nardelli *et al*., 2007). Collectively, these data underscore the importance of either translational or post-translational control in trypanosomatids.

The present study seeks to improve our understanding of signal events that might regulate the translation process in *T. brucei*. The study focuses on the trypanosome homologue of mammalian RACK1 (TbRACK1). RACK1 was initially identified as a signal anchor protein for protein kinase C and its targets (Ron *et al*., 1994). Since its initial discovery, the list of interacting proteins has grown considerably. It is now evident that RACK1 forms productive interactions with several kinases (Chang *et al*., 1998; McLeod *et al*., 2000; Stebbins and Mochly Rosen, 2001; Yaka *et al*., 2002), phosphatases (Mourton *et al*., 2001; Kiely *et al*., 2008), ion channels (Patterson *et al*., 2004; Onishi *et al*., 2006; Isacson *et al*., 2007), signal proteins (Koehler and Moran, 2001; Chen *et al*., 2005; Bolger *et al*., 2006; Hu *et al*., 2006; Zeller *et al*., 2007), receptors (Liliental and Chang, 1998) and proteins involved in gene expression (Chicurel *et al*., 1998; Angenstein *et al*., 2002; Ceci *et al*., 2003; Shor *et al*., 2003; Baum *et al*., 2004; Fomenkov *et al*., 2004; Gavin *et al*., 2006; Okano *et al*., 2006; Zhang *et al*., 2006). More recently, RACK1 has been identified as a new member of the eukaryotic translation machinery (Nilsson *et al*., 2004). Mass-spectroscopy studies identified RACK1 among the ribosomal proteins from *S. cerevisiae* (Link *et al*., 1999), *Arabidopsis* (Giavalisco *et al*., 2005) and humans (Yu *et al*., 2005). Cryo-electron microscopy localized RACK1 to the 40S head region near the mRNA exit pore where it makes direct contact with ribosomal RNA (Sengupta *et al*., 2004). The mRNA for mammalian RACK1 even shares the same control elements with other ribosomal transcripts; allowing them to be translationally controlled in a concerted way by signals involving PI3 kinase and mTOR (Loreni *et al*., 2005). Although one study with yeast suggests that RACK1 is found nowhere else in the cell except on ribosomes (Gerbasi *et al*., 2004), other studies with *S. cerevisae*, *S. pombe* and human cells indicate that RACK1 is found in both ribosomal and non-ribosomal fractions (Ceci *et al*., 2003; Shor *et al*., 2003; Baum *et al*., 2004; Zeller *et al*., 2007). The persistence of RACK1 on ribosomes under high salt conditions (Inada *et al*., 2002), the presence of RACK1 at a 1:1 ratio with other ribosomal proteins (Link *et al*., 1999) and its confirmed presence at high occupancy on isolated ribosomes by cryo-EM (Sengupta *et al*., 2004) indicate that most ribosomes in the cell contain RACK1.

RACK1 is important for ribosome function. Although not essential for cell viability, its deletion causes an increase in 40S and 60S subunits, a decrease in 80S subunits and formation of stalled initiation complexes, indicating a defect in the initiation step of translation (Chantrel *et al*., 1998). RACK1 has been shown to recruit activated protein kinase C to the ribosome and, through phosphorylation of eukaryotic initiation factor 6 (eIF6), allow the formation of a functional 80S complex (Ceci *et al*., 2003). RACK1 also affects the phosphorylation of other translation initiation factors, including eIF2a, eIF2Ø, eIF4A and the ribosome associated complex RAC (Valerius *et al*., 2007). Although RACK1 can repress global translation rates (Gerbasi *et al*., 2004), it also regulates the translation of specific transcripts. For example, polysomes prepared from yeast that have been depleted of RACK1 contain a reduced level of the *L25* transcript and lower levels of the L25 protein. No other mRNA analysed in the study was affected (Shor *et al*., 2003). When differential gel electrophoresis was used to measure protein levels in cells depleted of RACK1, It was shown that 27 proteins of 1500 were selectively upregulated and 3 proteins were significantly downregulated (Gerbasi *et al*., 2004). Because RACK1 also interacts with some membrane bound receptors (Liliental and Chang, 1998), it has been proposed that RACK1 may promote the docking of ribosomes to sites where local translation is required (Chicurel *et al*., 1998; Cox *et al*., 2003). In this regard, RACK1 may be critical for positional translation within the cell. Because no homologues of RACK1 have been identified in prokaryotes, it is likely that RACK1 performs eukaryotic-specific regulatory roles (Dresios *et al*., 2006).

In trypanosomes, a precise function for TbRACK1 has yet to be determined. Early studies indicated that the level of TbRACK1 transcripts became elevated as growth rates slowed. G0 arrested stumpy cells had a higher level of TbRACK1 mRNA than was found in rapidly dividing bloodstream form (BF) or procyclic form (PF) stages (Matthews and Gull, 1998). TbRACK1 mRNA also became elevated in cells undergoing apoptosis-like death (Welburn and Murphy, 1998). In *Leishmania*, disruption of the locus for LmRACK1 altered parasite viability and virulence (Kelly *et al*., 2003). Although TbRACK1 and LmRACK1 have each been associated with cell growth and/or infectivity, the pathways they regulate are unknown. We have shown that depletion of TbRACK1 with RNA interference (RNAi) generates cells that cannot complete cytokinesis. The cells contain several partial cleavage furrows, multiple nuclei and multiple flagella (Rothberg *et al*., 2006). This observation has led us to propose that TbRACK1 contributes towards the regulation of the final stage of mitosis (Rothberg *et al*., 2006). A similar observation was made in *C. elegans* where knockdown of RACK1 by RNAi generated defects in cleavage furrow ingression (Skop *et al*., 2004). However, the pathways RACK1 may use to affect cytokinesis or cell growth have not been identified. The spatial organization of TbRACK1 does not provide any clues about how it functions. It is found in both particulate and supernatant fractions, along the nuclear envelope, but is not associated with the cleavage furrow, ER or with the mitochondrion (Rothberg *et al*., 2006). A cytoplasmic distribution has also been reported for *Crithidia* CfRACK1 (Taladriz *et al*., 1999) and CPC2/RACK1 in *S. pombe* (McLeod *et al*., 2000). The following study develops the hypothesis that TbRACK1 recruits signal components to the ribosome and plays a role in the differential expression of trypanosome genes. The regulation of specific proteins may account for the correlation between TbRACK1 levels and cell growth and explain its ability to modulate cytokinesis. Although the hypothesis builds upon considerable precedence in other eukaryotic systems, it is complicated by current models of the trypanosome ribosome. The organization of the trypanosome ribosome was best delineated by a cryo-EM analysis of 80S monosomes from *T. cruzi* (Gao *et al*., 2005). Surprisingly, TcRACK1 was conspicuously absent, and the authors concluded that trypanosome ribosomes are fundamentally different from those in other eukaryotes. Here we test whether the absence of ribosomal RACK1 is a distinguishing feature of the trypanosome translation machinery. We demonstrate that TbRACK1 is found in protein complexes that contain eukaryotic elongation factor-1 A (eEF1A), and is present on monosomes and translating polysomes from *T. brucei* and *T. cruzi*. TbRACK1 dissociates from ribosomes under conditions of high salt or with low amounts of deoxycholate. When RNAi is used to deplete the cellular content of TbRACK1, the polysome profile changes in a manner that is consistent with stalled translation initiation. Additionally, a 30 kDa ribosomal protein becomes hyperphosphorylated on a Thr residue. As further evidence of a disruption in ribosome function, the growth of TbRACK1 deficient cells becomes hypersensitive to the translational inhibitor anisomysin. At subtoxic doses, anisomycin augments the effects of TbRACK1 RNAi by greatly increasing the percentage of cells exhibiting a cytokinesis defect. Other aspects of cell cycle progression appear to be unaffected, including nuclear division, flagellar assembly and motility. Collectively, these data demonstrate that TbRACK1 is a functional component of the trypanosome ribosome and demonstrate that cytokinesis is selectively inhibited when translation is disrupted.

## Results

### TbRACK1 associates with eEF1A

**Fig. 1:**
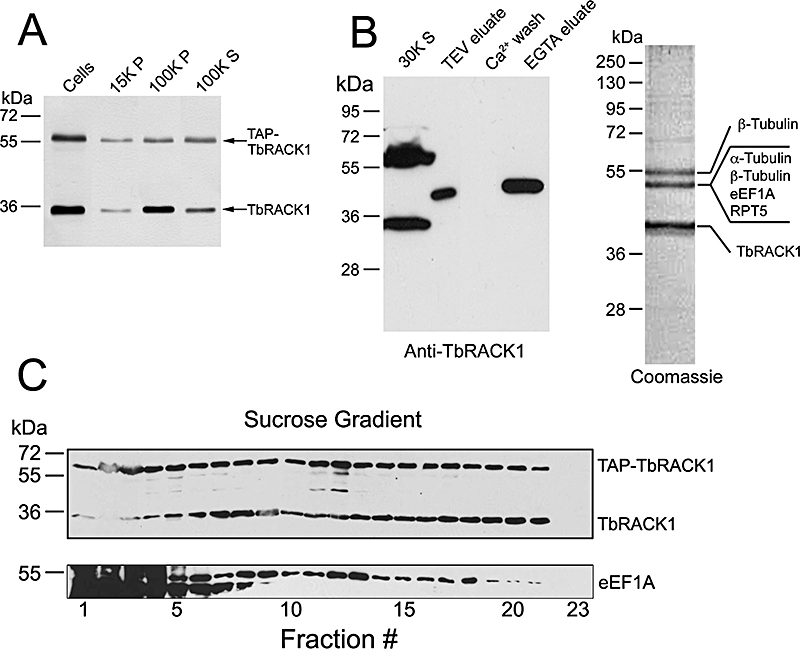
The identification of TAP-TbRACK1 binding partners. A. Expression of TAP-TbRACK1 and its distribution in cultured PF. A cloned PF cell line was produced that constitutively expressed the TAP-Tag fused to the amino terminus of TbRACK1. Cells were fractionated by centrifugation and each fraction was suspended in the original homogenate volume. Proteins were separated on a 12% polyacrylamide gel and analysed by Western blot with rabbit anti-TbRACK1 to evaluate the relative abundance of TAP-TbRACK1 (55.5 kDa) compared with endogenous TbRACK1 (34 kDa). B. Affinity purification of TAP-TbRACK1 and its associated proteins. In the left panel, Western blot with rabbit anti-TbRACK1 is used to identify TAP-TbRACK1 and endogenous TbRACK1 during the affinity purification steps. The TEV-protease eluted proteins from the IgG-Sepharose beads, while the Ca^2+^ wash and pooled EGTA eluates were from the calmodulin-Sepharose beads. A new band at 40 kDa was generated by the TEV-protease cleavage of the TAP-TbRACK1. The right panel is the Coomassie-stained gel of the EGTA eluate. The three predominant bands were excised and each band was analysed by LC MS/MS. Proteins that comprised each band are listed. C. TbRACK1, TAP-TbRACK1 and eEF1A cosediment on sucrose gradients. About 10^9^ log phase cells were disrupted with glass beads followed by the addition of 1.2% (v/v) Triton X-100. The 14 000 *g* supernatant was applied to a 12 ml linear sucrose gradient (15–50%) and centrifuged for 2.5 h at 170 000 *g* (avg). Fractions (0.5 ml each) were collected and proteins were precipitated with methanol/chloroform. Each fraction was separated by SDS-PAGE on 12% acrylamide gels, and probed with rabbit anti-TbRACK1 (upper panel) or with mouse anti-eEF1A (lower panel).

We have previously shown that cells become arrested midway through cytokinesis when RNAi is used to deplete the cellular content of TbRACK1 (Rothberg *et al*., 2006). To understand the mechanisms of growth arrest, the TbRACK1 binding partners were sought. A Tandem Affinity Purification Tag (TAP-Tag) (Rigaut *et al*., 1999) was fused to the 5′ end of full-length TbRACK1 and the construct was cloned into the constitutive expression vector pTSA.Hyg2 (Sommer *et al*., 1992). A PF cell line was established that stably expressed the TAP-TbRACK1 construct. Western blot with anti-TbRACK1 antibodies recognized both the endogenous TbRACK1 (34 kDa) and the larger TAP-TbRACK1 (55.5 kDa). A crude cellular fractionation revealed that, similar to the endogenous protein, the TAP-TbRACK1 was present in each of the pellet and supernatant fractions (Fig. 1A). A low speed supernatant fraction (14 000 *g*) was further fractionated on sucrose gradients and endogenous TbRACK1 comigrated with TAP-TbRACK1 (Fig. 1C). Proteins that bound to TAP-TbRACK1 were initially separated on an immunoglobulin affinity column and released with TEV protease (Fig. 1B; TEV eluate lane). The protealytically cleaved TAP-TbRACK1 (40 kDa) was detected in the elution fraction but not the native TbRACK1 (34 kDa). These data indicate that endogenous TbRACK1 does not form dimers with the TAP-TbRACK1. The TAP-TbRACK1 was then bound to a calmodulin-Sepharose affinity column, washed until no more protein was detected (Ca^2+^ wash lane) and eluted with EGTA. The cleaved TAP-TbRACK1 was detected by Western blot as a 40 kDa band. When this final elution fraction was evaluated for proteins by Coomassie stain, three predominant bands at 54, 50 and 40 kDa were consistently observed in three separate preparations (Fig. 1B). Each band was excised and analysed by tandem MS. The 54 kDa band corresponded to β-tubulin (13 fragments), while the 40 kDa band corresponded to TEV-cut TAP-TbRACK1 (9 fragments). The 50 kDa band was heterogeneous and contained four proteins of ∼50 kDa including α-tubulin (11 fragments), β-tubulin (9 fragments), eEF1A (5 fragments) and proteasome regulatory ATPase subunit 5 (RPT5; 2 fragments).

**Fig. 2:**
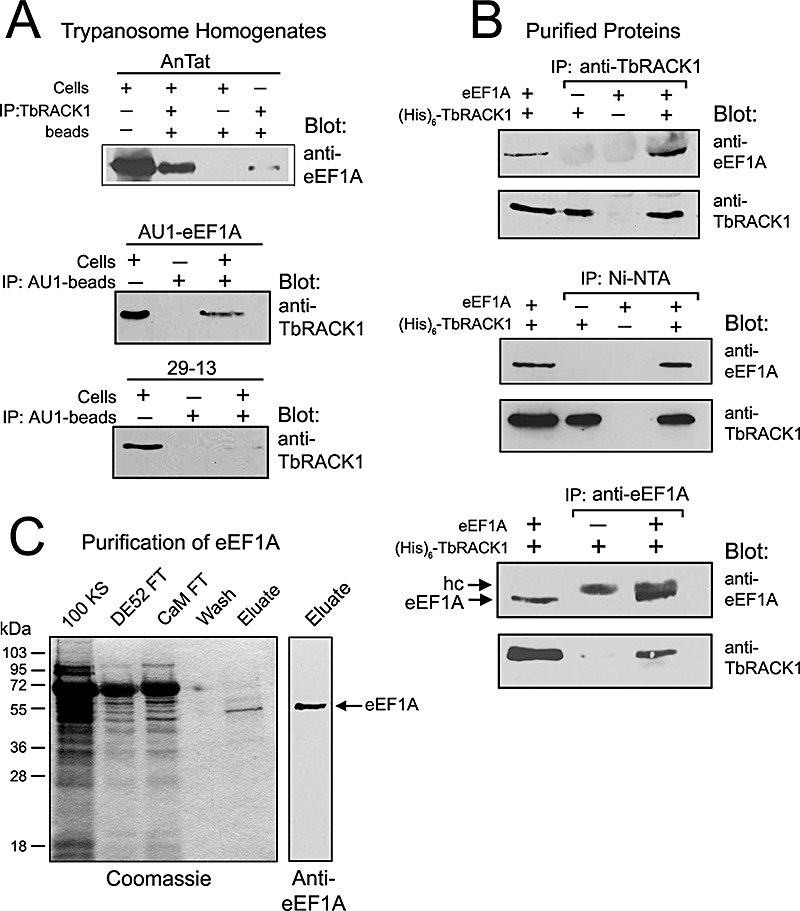
TbRACK1 and eEF1A are binding partners. A. TbRACK1 associates with eEF1A in cell homogenates. Upper panel: Wild-type PF (AnTat1.1) were gently lysed, and the 10 000 *g* supernatant fraction was used for immunoprecipitation pull-downs. Each tube was incubated with combinations of cell homogenates, rabbit anti-TbRACK1 and protein A beads, as indicated. Western blot analysis was with mouse anti-eEF1A. In each panel, the input is shown in the left lane. Middle panel: 29-13 PF cells were transformed with AU1-eEF1A in pLEW100. After 3 days induction with 1 μg ml^−1^ tetracycline, cell homogenates were precipitated with anti-AU1 beads and the pull-down of TbRACK1 was monitored. Lower panel: Homogenates of parental 29-13 cells were precipitated with anti-AU1 beads and the pull-down of TbRACK1 was monitored. B. Purified native eEF1A and (His)_6_-TbRACK1 coimmunoprecipitate. Reactions contained different combinations of purified native eEF1A (10 μg) and recombinant (His)_6_-TbRACK1 (8 μg), as indicated. Anti-TbRACK1 pulled down eEF1A only when (His)_6_-TbRACK1 was present in the assay (upper panel). Ni-NTA pulled down eEF1A only when (His)_6_-TbRACK1 was present in the assay (middle panel). Anti-eEF1A pulled down (His)_6_-TbRACK1 only when eEF1A was present in the assay (lower panel). The antibody heavy chain (hc) is visible in the pull-down with anti-eEF1A (lower panel). The input is shown in the left lanes. C. Purification of native eEF1A from trypanosome homogenates. Coomassie-stained gel of the eEF1A purification. Individual lanes contain the 100 K supernatant; DE-52 flow through; calmodulin-Sepharose flow through; final wash; and EGTA eluate from the calmodulin-Sepharose column. Adjacent panel is the Western blot of the final eluate.

**Fig. 3:**
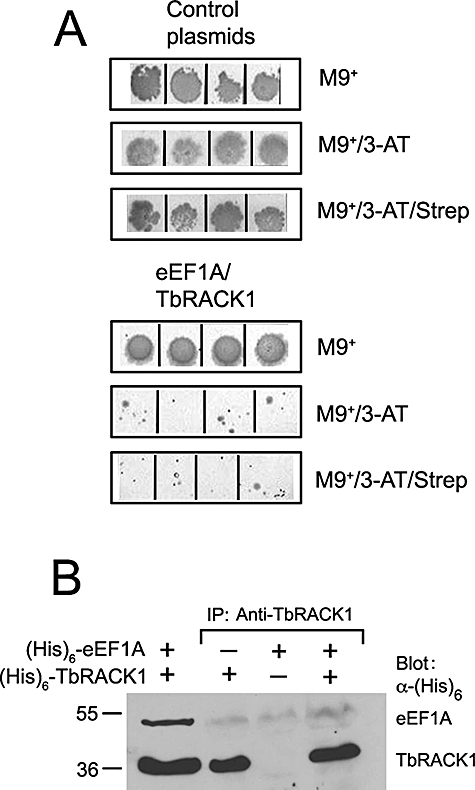
Bacterially expressed recombinant eEF1A does not interact with TbRACK1. A. eEF1A and TbRACK1 fail to interact by bacterial two-hybrid. BactrioMatch II validation reporter competent cells (Stratagene) were co-transformed with bait and prey, and then plated on non-selective M9^+^ plates. Ten colonies from the M9^+^ non-selective plates were suspended in liquid culture and re-plated onto either: M9^+^ medium; M9^+^ with 5 mM 3-AT; or M9^+^ with 5 mM 3-AT and 12.5 μg ml^−1^ streptomycin. For the sake of space, only four colonies are shown. Control plasmids were purchased from Stratagene, and consisted of bait (pBT-LGF2) and prey (pTRG-GAL1 1^P^). B. Bacterially expressed recombinant (His)_6_-eEF1A does not interact with recombinant (His)_6_-TbRACK1. Binding assays contained combinations of 8 μg (His)_6_-TbRACK1 and 8 μg (His)_6_-eEF1A, as indicated. Immunoprecipitation was with anti-TbRACK1 and proteins in the pull-down were detected with anti-His_6_ antibodies. eEF1A is not pulled down by these procedures. The input is in the left lane.

Here we focus on the association between TbRACK1 and eEF1A because interactome analyses from *S. cerevisiae* or *C. elegans* had not previously identified this pair and because their association has implications towards translational regulation. The TAP-TbRACK1 has the potential to misidentify binding partners because it may interact through the tag region, fold inappropriately or traffic to the wrong location in the cell. We employed sucrose gradient centrifugation to verify that protein complexes containing eEF1A also contained TbRACK1 (Fig. 1C). A monoclonal antibody against trypanosome eEF1A was used (Kaur and Ruben, 1994), and it recognized a protein of 50 kDa that comigrated throughout the gradient with TbRACK1 and TAP-TbRACK1. A smaller fragment cross-reacted with the antibody, and its distribution was limited to the top fractions of the gradient. To establish whether TbRACK1 was in a complex with eEF1A, wild-type PF cells (derived from AnTat1.1 BF) were gently lysed and the cell homogenate was immunoprecipitated with rabbit antibodies against TbRACK1 (Fig. 2A; upper panel). Specific coprecipiation of eEF1A was observed. For the reciprocal immunoprecipitation, 29-13 PF were transformed with AU1 epitope tagged eEF1A. The anti-AU1 beads immunoprecipitated TbRACK1 in cells transformed with the AU1-eEF1A construct (Fig. 2A, middle panel), but not in parental 29-13 cells (Fig. 2A, lower panel). To test whether the binding between TbRACK1 and eEF1A was direct, native eEF1A was purified from trypanosome homogenates (Fig. 2C) and incubated with recombinant (His)_6_-TbRACK1 from *E. coli*. Reciprocal pull-down assays were performed with the purified proteins (Fig. 2B). Anti-TbRACK1 or Ni-NTA agarose each precipitated native eEF1A, but only when (His)_6_-TbRACK1 was present in the reaction (upper and middle panels). Conversely, anti-eEF1A precipitated (His)_6_-TbRACK1 only when native eEF1A was present in the reaction (lower panel). Because previous interactome studies from *S. cerevisiae* or *C. elegans* did not identify RACK1 and eEF1A as direct binding partners, we assessed whether recombinant eEF1A retains the ability to bind TbRACK1. When protein interactions were evaluated either by bacterial two-hybrid, or by immunoprecipitation of purified recombinant proteins, no direct binding was observed (Fig. 3A and B). Therefore, only native eEF1A was able to bind recombinant TbRACK1.

### TbRACK1 associates with trypanosome monosomes

The appearance of eEF1A among the TbRACK1 binding partners caused us to re-evaluate the current structural model of the trypanosome ribosome. This model was generated from an elegant cryo-EM examination of purified monosomes from *T. cruzi* (Gao *et al*., 2005). The analysis revealed fundamental differences between ribosomes from trypanosomes and those from a range of eukaryotes, including yeast, plants and humans. Notable among the differences was the absence of TcRACK1. Here we use different methods to determine whether TbRACK1 associates with trypanosome ribosomes. If this association occurs, then TbRACK1 has the potential to regulate translation through the recruitment of signal proteins. If this association does not occur, then the trypanosome ribosome is fundamentally different due to the absence of TbRACK1.

**Fig. 4:**
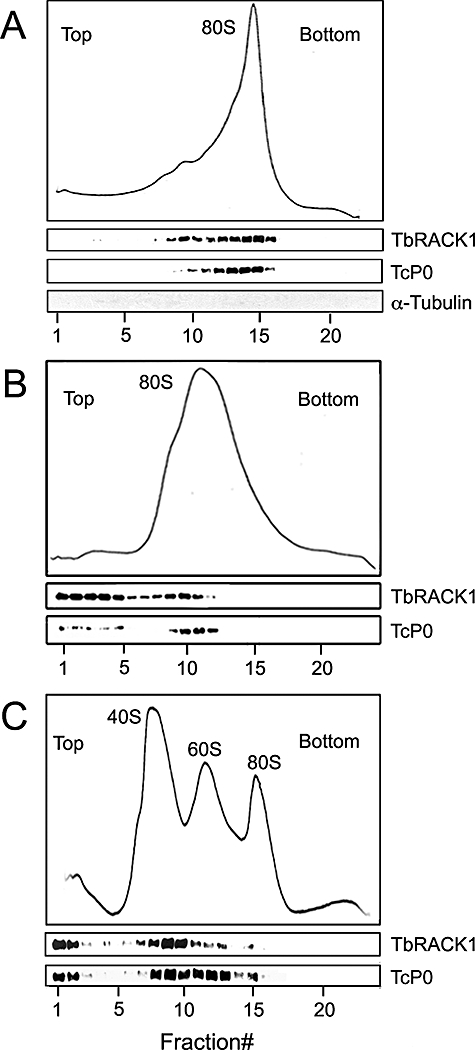
TbRACK1 is a component of trypanosome monosomes, and can be released with high concentrations of NaCl or 0.2% deoxycholate. A. Purification of monosomes from wild-type PF. Monosomes were partially purified through a step gradient of 20%/40% sucrose, and separated on a linear gradient of 5–25% sucrose. The OD_254_ was continuously recorded and 0.5 ml fractions were collected. Proteins in each fraction were precipitated by methanol/chloroform and separated by SDS-PAGE. Western blot was used to detect TbRACK1, TcP0 or α-tubulin. B. The crude ribosome fraction was incubated with 0.7 M NaCl for 30 min prior to loading on the sucrose gradient. C. The crude ribosome fraction was incubated with 0.2% deoxycholate for 45 min prior to loading on the sucrose gradient.

To test this hypothesis, 80S monosomes were prepared from log phase cells using a modification of previously described procedures (Brecht and Parson, 1998; Rodríguez-Gabriel *et al*., 2000). In this procedure, ribosomes were centrifuged through a sucrose cushion prior and then fractionated on a 5–25% linear sucrose gradient (Fig. 4A). Fractions from the sucrose gradient were assessed for specific proteins by Western blot analysis (Fig. 4A). The monosome peak was identified with antibodies against TcP0 (Skeiky *et al*., 1993). TcP0 belongs to the P-protein family. P-proteins form a long stalk on the 60S subunit of eukaryotic ribosomes. A protein interaction map of the *T. cruzi* P-proteins has been determined (Ayub *et al*., 2005). Antibodies against TcP0 demonstrated that the large protein peak in our chromatogram was comprised of trypanosome monosomes. TbRACK1 was also found in the same monosome peak. It is not likely that TbRACK1 is in association with contaminating material. Membrane bound compartments were eliminated when the sample was centrifuged through a high-density sucrose cushion, while the absence of α-tubulin indicates that cytoskeletal contamination is also absent. The interaction between TbRACK1 and monosomes was disrupted with 0.7 M NaCl (Fig. 4B). This result is consistent with the situation in *S. pombe*, where RACK1 also releases from the ribosome in the presence of high salt concentrations (Shor *et al*., 2003). Finally, when treated with 0.2% deoxycholate the 80S monosome became unstable (Fig. 4C). It divided into its 40S and 60S components. TbRACK1 was released to the top of the gradient and the remaining 80S monosome fraction was devoid of TbRACK1. Some of the TbRACK1 remained associated with the 40S subunit.

### TbRACK1 binds to ribosomes while they are actively engaged in translation

**Fig. 5:**
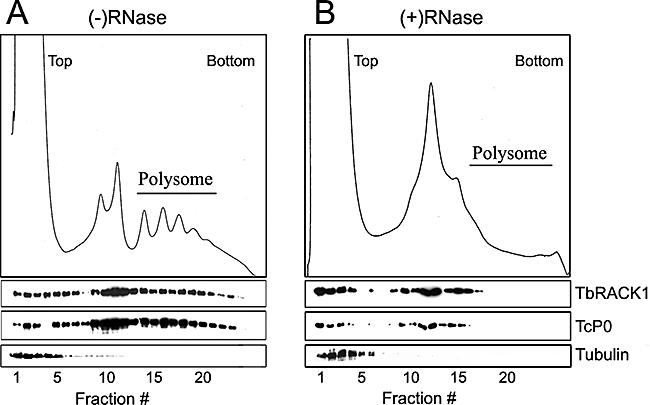
Identification of TbRACK1 in polysome preparations from PF. A. Polysomes were harvested from a 15–50% linear sucrose gradient. The OD_254_ was continuously recorded and 0.5 ml fractions were collected. Proteins in each fraction were precipitated by methanol/chloroform and separated by SDS-PAGE. Western blot was used to detect TbRACK1, TcP0 or α-tubulin. B. Polysomes were incubated with 0.5 mg ml^−1^ RNase A for 30 min prior to separation on the 15–50% linear sucrose gradient.

We sought to determine if TbRACK1 remains associated with ribosomes while they are involved in translation. To accomplish this goal, polysomes were stabilized with 100 μg ml^−1^ cyclohexamide and isolated on sucrose gradients. When the gradients were harvested, a classic polysome profile was observed (Fig. 5A). The 60S ribosomal marker protein TcP0 was in the monosome and polysome fractions. TbRACK1 had a similar distribution to TcP0 and also was found in non-ribosomal fractions at the top of the sucrose gradient. Tubulin did not extend far into the gradient, precluding the possibility that the distribution of TbRACK1 in the polysome fraction was dependent upon its association with cytoskeletal elements. To further verify that TbRACK1 was associated with polysomes, the mRNA thread holding the polysome together was cut with RNAse (Fig. 5B). Following this treatment, the polysome pattern was disrupted and both TcP0 and TbRACK1 shifted towards the top of the gradient. The distribution of tubulin was unchanged. Altogether, these data demonstrate that TbRACK1 associates with monosomes and stays with the ribosomes when they assemble into actively translating polysomes.

### TbRACK1 binds to polysomes from BF of *T. brucei* and epimastigotes of *T. cruzi*

**Fig. 6:**
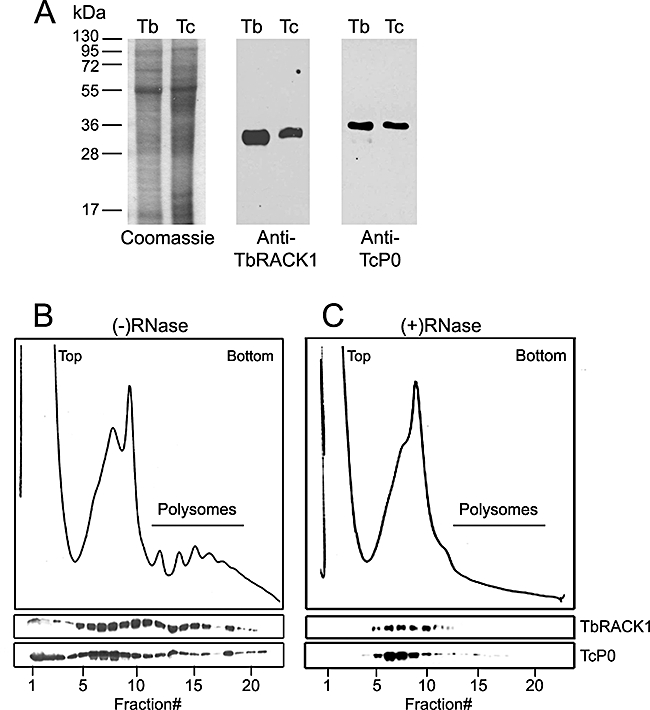
Polysomes from *Trypanosoma cruzi* epimastigotes contain TcRACK1. A. Antibodies detect RACK1 and P0 from *T. brucei* PF and *T. cruzi* epimastigotes with high specificity. Whole-cell homogenates (15 μg) were separated by SDS-PAGE and were analysed by Western blot with antibodies against TbRACK1 or TcP0. B. Polysomes were harvested from a 15–50% linear sucrose gradient. The OD_254_ was continuously recorded and 0.5 ml fractions were collected. Proteins in each fraction were precipitated by methanol/chloroform and separated by SDS-PAGE. Western blot was used to detect TcRACK1, TcP0 or α-tubulin in each fraction. C. Polysomes were incubated with 0.5 mg ml^−1^ RNase A for 30 min prior to separation on the 15–50% linear sucrose gradient.

So far, we report that TbRACK1 associates with monosomes and polysomes from PF trypanosomes. During the transformation process from insect to mammalian host, changes in gene expression occur. Here we evaluate whether the association of TbRACK1 with polysomes is developmentally regulated. TbZFP3 is an example of a protein that is found in polysome fractions from PF but is absent from polysomes of BF (Paterou *et al*., 2006). Polysome fractions from BF strain M110 also contain TbRACK1 (Fig. S1). Our results differ from those obtained with *T. cruzi* monosomes. This may result from fundamental differences between ribosomes from *T. cruzi* and *T. brucei* or reflect differences in methods. To resolve this issue, polysomes were analysed from epimastigotes of *T. cruzi*. Our antibody reagents were suitable for this analysis, as they recognized a single band from whole-cell homogenates of either *T. brucei* or *T. cruzi* (Fig. 6A). Both TcP0 and TcRACK1 were components of the polysome fractions (Fig. 6B). These proteins shifted their distribution when polysomes were disrupted with RNAse (Fig. 6C). Therefore, RACK1 homologues are found in association with polysomes from PF and BF of *T. brucei*, and epimastigotes of *T. cruzi*.

### Depletion of TbRACK1 by RNAi alters properties of trypanosome polysomes

**Fig. 7:**
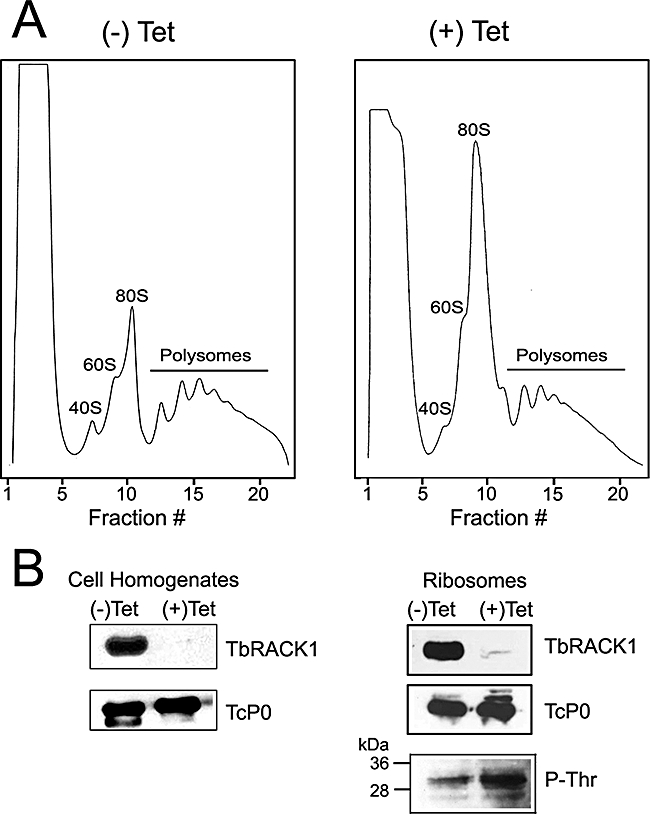
Conditional knockdown of TbRACK1 changes the polysome profile and increases phosphorylation of a ribosomal protein in *T. brucei* PF. A. Polysome profiles were obtained from 1 × 10^9^ TbRACK1 RNAi cells, either without induction of RNAi (−Tet), or after induction of RNAi (+Tet) for a 4 day period. Compared with uninduced cells and control wild-type cells, polysomes from TbRACK1-depleted cells had an increased peak height of the 80S peak relative to the polysomes and a decreased average length of the polysome profile. B. The effect of TbRACK1 RNAi on phosphorylation of a 30 kDa ribosomal protein. After 4 days of RNAi induction, the level of TbRACK1 decreased in whole-cell homogenates, as determined by Western blot (left panels). Purified ribosomes from these cells were also depleted of TbRACK1 (right panels). The level of phosphorylated threonine in a 30 kDa protein increased. TcP0 was used as a loading control. Molecular weight markers in kDa are shown for the phospho-threonine blot.

To determine whether the association of TbRACK1 with ribosomes has functional consequences, RNAi was used to deplete PF of TbRACK1. Cells were stably transformed with plasmid pZJM containing 503 bp of TbRACK1 between dually opposed T7 promoters, as described by us previously (Rothberg *et al*., 2006). Western blot revealed that protein levels of TbRACK1 decreased over a 4 day period following induction of RNAi with 1 μg ml^−1^ tetracycline (Fig. 7B). Polysomes were prepared from 1 × 10^9^ PF (Fig. 7A). Without the induction of RNAi (−Tet), the polysome profile was similar to that produced by wild-type PF in which the height of the 80S monosome peak was two- to threefold higher than the polysome peaks (compare Figs. 5A and 7A). When TbRACK1 was depleted by RNAi (+Tet), polysomes still formed but the relative height of the 80S monosome peak was consistently larger and the number of polysome peaks was consistently reduced (Fig. 7A). A similar result was obtained in yeast following downregulation of the translation initiation factor eIF3-p39 (Naranda *et al*., 1997). The inefficient translation initiation resulted in an accumulation of 80S monosomes, while completion of the elongation step, led to fewer polysome peaks. Because yeast RACK1 affects the phosphorylation state of several initiation factors (Valerius *et al*., 2007), we purified ribosomes from control and TbRACK1-depleted cells (Fig. 7B). The gel lanes were standardized to contain the same amount of the 60S ribosomal marker TcP0. After induction of RNAi with tetracycline, the amount of ribosome-associated TbRACK1 is greatly reduced. Under these conditions, antibodies against phosphothreonine consistently detected a protein of around 30 kDa that became hyper-phosphorylated (Fig. 7B). Antibodies against phosphoserine or phosphotyrosine did not detect any changes (data not shown). The increased phosphorylation state of the 30 kDa protein might occur if TbRACK1 inhibits kinase activity, or if TbRACK1 is required to recruit a phosphatase to the ribosome.

### The translation inhibitor anisomycin works synergistically with TbRACK1 RNAi to arrest cell growth and generate the distinctive cytokinesis phenotype

**Fig. 8:**
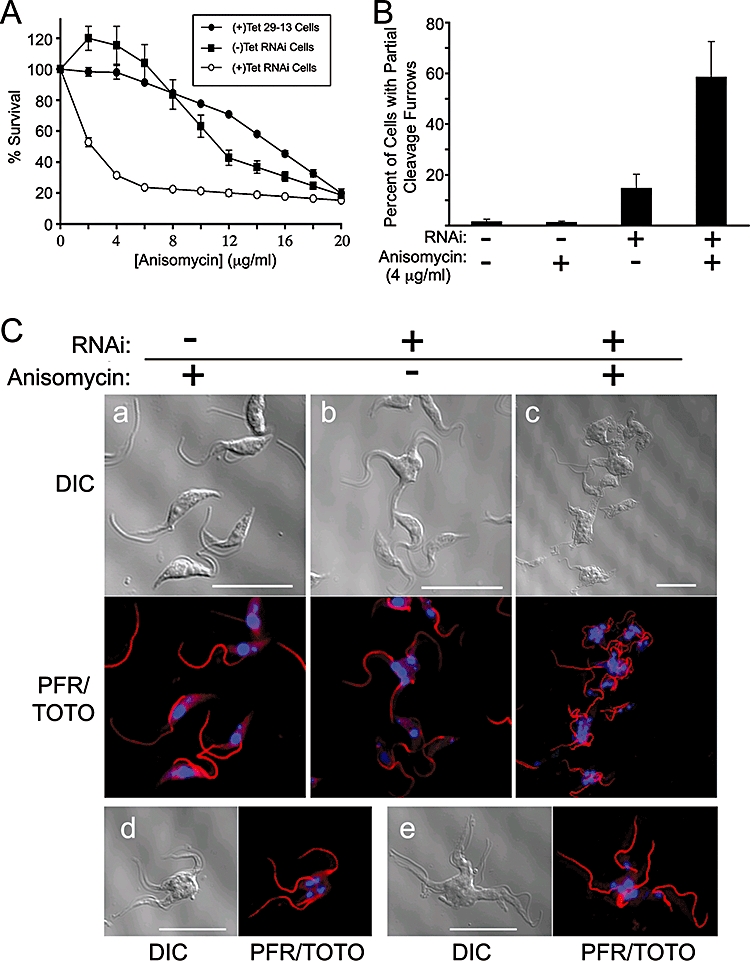
TbRACK1 RNAi cells are hypersensitive to anisomycin. A. Anisomycin affects growth of the TbRACK1 RNAi cells. A three-times cloned TbRACK1 RNAi cell line and the 29-13 parental cell line were grown for a 4 day period either without tetracycline or with 1 μg ml^−1^ tetracycline, as indicated. Increasing concentrations of anisomycin were added to the medium during the final 48 h. Cell density was recorded at the end of the 4 day period, and the value without anisomycin was set at 100%. Without anisomycin, the parental 29-13 cells grew to a density of 2.2 ± 0.08 × 10^7^ cells ml^−1^, the RNAi cells without tetracycline grew to 1.9 ± 0.2 × 10^7^ cells ml^−1^ while the RNAi cells with tetracycline grew to a density of 0.7 ± 0.01 × 10^7^ cells ml^−1^. Each point is the average ± SE of two independent experiments. B. Phenotypic changes in trypanosomes after treatment with subtoxic levels of anisomycin. Cells were treated with combinations of 1 μg ml^−1^ tetracycline for a total of 4 days and 4 μg ml^−1^ anisomysin was added during the final 48 h, as indicated. The cells were viewed by DIC microscopy and at least 200 cells were scored for partial cleavage furrow ingression. Each bar is the average ± SE of two independent experiments. C. Phenotypes of cells treated with subtoxic levels of anisomycin. Cells from the experiment in (B) were fixed, permeabilized and stained with the long wavelength laser dye TOTO (DNA) and with rabbit anti-PFR (flagella). (a) 4 μg ml^−1^ anisomycin alone; (b) TbRACK1 RNAi alone; (c) TbRACK1 RNAi with 4 μg ml^−1^ anisomycin (low magnification). (d)–(e) are higher magnification of the TbRACK1 RNAi cells after treatment with 4 μg ml^−1^ anisomycin. In each panel, the bar is 15 μm.

Anisomycin is an inhibitor of the transpeptidylation reaction of translation. An increased sensitivity of cell growth to translational inhibitors has been used by others to implicate proteins of unknown function in the translation process (Nelson *et al*., 1992; Spence *et al*., 2000). Therefore, cells depleted of TbRACK1 by inducible RNAi were compared with control cultures for their sensitivity to anisomycin. The control cultures were either the uninduced TbRACK1 RNAi cells (−Tet), or parental 29-13 cells plus tetracycline. Cultures were allowed to grow for 2 days in the presence or absence of tetracycline. The cells were diluted to a density of 1 × 10^6^ cells ml^−1^ and treated with variable amounts of anisomycin. At day 4, cell density was determined (Fig. 8A). Growth in the absence of anisomycin was set at 100%. Cells that were depleted of TbRACK1 exhibited increased sensitivity to anisomycin, with IC50 of 3 μg ml^−1^. By contrast, neither of the control cultures was affected by anisomycin at 3 μg ml^−1^. Instead, the uninduced RNAi cells or parental 29-13 cells had IC50 values of 11 and 16 μg ml^−1^ respectively (Fig. 8A). These data demonstrate that neither the pZJM.TbRACK1 plasmid nor treatment with tetracycline was sufficient on its own to sensitize cell growth to anisomycin.

During the cell counts, it was noted that the growth-arrested cells had a specific cell cycle defect. The cells were able to initiate cytokinesis but arrested after partial cleavage furrow ingression (Fig. 8B and C). As reported by us previously, when depleted of TbRACK1 by conditional knockdown, the postmitotic cells initiated cytokinesis, but became arrested partway through the process (Rothberg *et al*., 2006). Reiterations of mitosis, flagellar assembly and partial cytokinesis generated cells with multiple nuclei, multiple flagella and multiple partially formed cleavage furrows (Rothberg *et al*., 2006). Here we show that 4 μg ml^−1^ anisomycin was non-toxic to uninduced cells (Fig. 8A; −Tet) and that these cells had a normal morphology (Fig. 8B and C). The population was primarily comprised of cells with 1 nucleus and 1 kinetoplast (1N1K) and dividing cells had normal postmitotic positioning of kinetoplasts and nuclei (KNKN). To deplete cells of TbRACK1, RNAi was induced with tetracycline over a 4 day period. At the end of 4 days, approximately 15% of cells developed the distinctive phenotype characteristic of a cytokinesis defect (Fig. 7B and C). When a subtoxic dose of anisomycin (4 μg ml^−1^) was added during the final 2 days of RNAi induction, the number of abnormal cells increased to 60% of the population (Fig. 7B). Moreover, the 48 h exposure to this subtoxic dose of anisomycin exacerbated the severity of the cytokinesis phenotype (Fig. 7C and D). The cells had two or more partial cleavage furrows, multiple nuclei and multiple flagella; a phenotype which is characteristic of a postmitotic defect in cleavage furrow ingression (Fig. 7B–D). Cells also lost polarity as a consequence of this long-term exposure to anisomycin. To understand the process of cleavage furrow ingression, earlier time points were examined (see below).

**Fig. 9:**
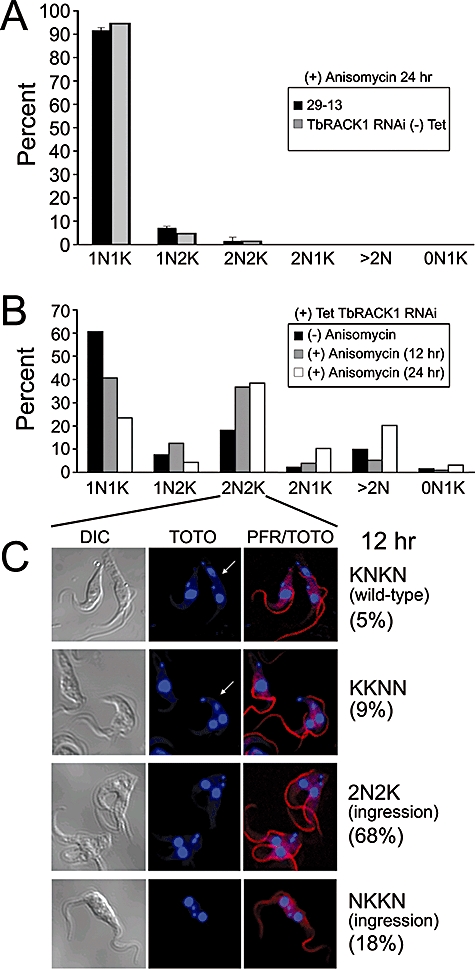
TbRACK1 depletion works synergistically with a subtoxic dose of anisomycin to block cleavage furrow ingression during cytokinesis. A. Anisomycin at 4 μg ml^−1^ does not affect cell cycle progression of parental 29-13 cells or the TbRACK1 RNAi cells without induction of RNAi (−Tet). The cells were treated for 24 h with anisomycin, fixed with paraformaldehyde, stained with DAPI and scored for nuclei (N) and kinetoplasts (K). The majority of cells are 1N1K. Shown is the average ± SE for two independent experiments. B. The effects of 4 μg ml^−1^ anisomycin on cell cycle progression after depletion of TbRACK1 with RNAi (+Tet). Early time points after treatment with anisomysin (12 h or 24 h). The subtoxic dose of anisomycin caused cells with 2N2K and > 2N to accumulate within 12 h of treatment. C. The cytokinesis defect begins with an abnormal configuration of kinetoplasts and nuclei. Cells were fixed with paraformaldehyde, permeabilized and stained with TOTO (DNA) and rabbit anti-PFR (flagella). The 2N2K cells were evaluated as a population. The cells were postmitotic and 14% of them had not initiated cytokinesis (sum of the upper two panels) while 64% of the cells had a partial cleavage furrow (sum of the lower two panels). Cells were classified as having a wild-type configuration (KNKN; in which the pattern of kinetoplasts and nuclei is described starting from the posterior end of the cell), misaligned nuclei and kinetoplasts (KKNN), partial cleavage ingression (2N2K ingression) and advanced cleavage ingression (NKKN ingression). The number in parentheses is the percentage of the total 2N2K population represented by a specific phenotype.

In *T. brucei*, a wide range of unrelated proteins can secondarily affect cytokinesis (Reviewed in Hammarton *et al*., 2007). The situation arises because the initiation of cytokinesis is generally dependent upon multiple cellular events, such as the duplication and segregation of single-copy organelles. To better understand the process by which anisomycin affects cleavage furrow ingression, the anisomycin-treated cells were monitored at early time points. The division of kinetoplast DNA (kDaNA) and nuclei were used as cytological markers for cell cycle progression (Woodward and Gull, 1990). In the G1 phase of the cell cycle, cells have a single kinetoplast (K) and single nucleus (N). The kinetoplast divides within the nuclear S phase producing cells with 1N2K. Post-mitotic cells have a configuration of 2N2K. By itself, the addition of 4 μg ml^−1^ anisomycin for a 24 h period was without effect on cell cycle progression of control 29-13 cells or uninduced TbRACK1 RNAi cells (Fig. 9A). In Fig. 9B, RNAi for TbRACK1 was induced with tetracycline for 4 days total. Anisomycin was either omitted, or was added during the last 12 or 24 h as indicated (Fig. 9B). After just 12 h exposure to anisomycin, 40% of the TbRACK1-depleted cells were in a postmitotic state with 2 nuclei and 2 kinetoplasts (2N2K; Fig. 9B). The 2N2K cells fell into two broad categories; 14% of these cells had not yet initiated cytokinesis (Fig. 9C; sum of the top two panels), while 86% were stalled in the process of cleavage furrow ingression (Fig. 9C; sum of the bottom two panels). Of the cells that had not yet initiated cytokinesis, the majority failed to align their duplicated nuclei and kinetoplasts properly. As a consequence, they had an unusual KKNN configuration instead of the wild-type KNKN configuration. Once cleavage furrow ingression began, it became stalled just anterior to the paired nuclei in 68% of all 2N2K cells. In 18% of the 2N2K cells, the cleavage furrow arrested just posterior to the nuclei (Fig. 9C). These latter cells had an unusual NKKN configuration. Collectively, these data demonstrate that depletion of TbRACK1 predisposes cells to subtoxic levels of anisomycin. The cells develop a specific defect in kinetoplast alignment which could result in a disruption of cytokinesis. Although anisomycin is a general inhibitor of translation, the phenotype we report is quite specific. The trypanosomes are still metabolically active (motile), undergo continued mitosis (multinucleate), replicate their kinetoplasts (multiple K) and assemble new flagella (multiflagellate). The data demonstrate that anisomycin and TbRACK1 RNAi behave synergistically to regulate the last stage of cell division.

**Fig. 10:**
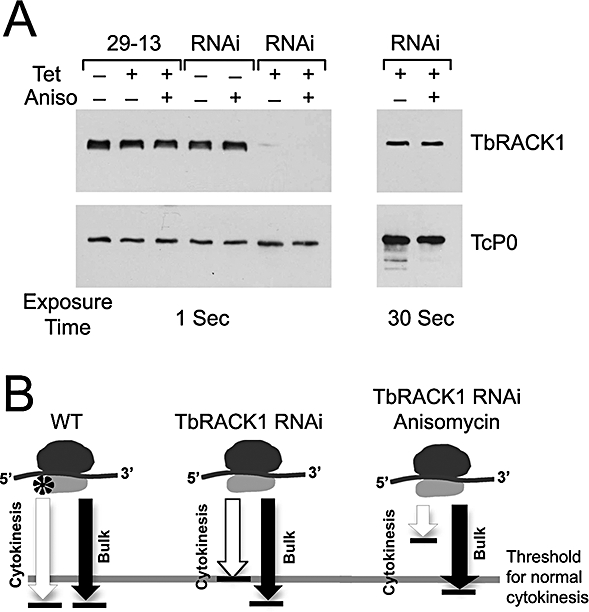
Translational inhibition and cytokinesis. A. Anisomycin does not lower the levels of TbRACK1 in control cells or TbRACK1 depleted cells. Cell cultures were treated with combinations of tetracycline (1 μg ml^−1^) for 4 days and with anisomycin (4 μg ml^−1^ for 24 h), as indicated. Whole-cell homogenates (10 μg per lane) were separated by SDS-PAGE, and the expression levels of TbRACK1 or TcP0 were determined by densitometry of Western blots. A longer exposure was required to detect TbRACK1 in the RNAi cells +Tet (right panels). Blots similar to this one were quantified by densitometry and the level of TbRACK1 in each sample was expressed as the ratio of IDV values for TbRACK1:TcP0. B. A model to explain the synergy between anisomycin and depletion of TbRACK1. We propose that in wild-type cells (WT), translation is sufficient to sustain cell viability and cytokinesis. When expression of TbRACK1 is decreased with RNAi, the expression level of a cytokinesis protein approaches a critical threshold, while bulk translation continues. The translational inhibitor anisomycin at 4 μg ml^−1^ has no effect on cell cycle progression of control cultures, but we propose that it lowers expression of the cytokinesis protein below a critical threshold.

One hypothesis to account for these data is that TbRACK1 regulates translation of a cell cycle protein and that anisomycin further inhibits its translation below a threshold level. Alternatively, anisomycin could merely help to lower the level of TbRACK1 beyond that achieved with RNAi alone. To test the latter hypothesis, the effects of anisomycin on expression levels of TbRACK1 were measured (Fig. 10A). Equivalent amounts of cell homogenates (10 μg lane^−1^) were separated by SDS-PAGE, and blots were probed with anti-TbRACK1 or TcP0 as a loading control. Protein was quantified by densitometry and the level of TbRACK1 in each sample was defined as the ratio of IDV values for TbRACK1:TcP0. This ratio was unaffected by anisomycin in either control 29-13 cells or in the TbRACK1 RNAi cells whether or not tetracycline was present. In the RNAi cells treated with both anisomycin and tetracycline, the level of TbRACK1 was 97 ± 5% of the level with just tetracycline alone (average ± SE; *n* = 6). Altogether, these data demonstrate that the phenotypic changes produced by anisomycin are not a consequence of lowering expression levels of TbRACK1 in the RNAi-induced cells. We propose that TbRACK1 regulates translation of a specific cell division protein (Fig. 10B). Depletion of TbRACK1 by RNAi decreases translation of this protein to a threshold level and cytokinesis is inhibited in 15% of cells. Anisomycin at 4 μg ml^−1^ further lowers expression of this protein below a threshold level, accounting for the observation that 60% of cells exhibit a disruption in cleavage furrow ingression.

Overall, we provide the first demonstration from any species that a RACK1 homologue binds directly with eEF1A. This is also the first report to demonstrate that TbRACK1 is associated with trypanosome monosomes and polysomes. The depletion of TbRACK1 by RNAi alters the polysome profile and causes increased phosphorylation of a 30 kDa ribosomal protein. The depletion of TbRACK1 also disrupts cell division by leading to a misalignment of kinetoplasts and nuclei in postmitotic cells; preventing complete cleavage furrow ingression during cytokinesis. Sub-toxic levels of the translational inhibitor anisomycin work synergistically with TbRACK1 depletion to inhibit cytokinesis. The effect does not result from further changes in the expression level of TbRACK1. We conclude that continued translation is required for normal cytokinesis to proceed.

## Discussion

### TbRACK1 forms a complex with eEF1A

A wide variety of signal proteins have been identified in *T. brucei*, but these have yet to be assembled into a simple regulatory pathway or interacting network. We began to study the anchor protein TbRACK1 as a tool to investigate trypanosome signal pathways. It had previously been established that expression levels of TbRACK1 inversely correlated with growth rate and that the *Leishmania* homologue LmRACK1 was required for infectivity in the mammalian host (Kelly *et al*., 2003). We demonstrated that depletion of TbRACK1 by RNAi caused a significant proportion of cells to arrest partway through cytokinesis (Rothberg *et al*., 2006). Interestingly, a similar result was reported with zygotes from *C. elegans*. As observed with trypanosomes, the zygotes were able to initiate cytokinesis in the absence of RACK1; however, the cleavage furrow arrested after the onset of ingression and then dissipated (Skop *et al*., 2004). The identification of RACK1 among the proteins in the mid-body of synchronized CHO cells led us to hypothesize that TbRACK1 might form ternary signal complexes to activate proteins involved in furrow ingression. In species other than trypanosomes, a separate hypothesis derives from the observation that RACK1 homologues are associated with ribosomes, ribonucleoprotein complexes and transmembrane receptors (Angenstein *et al*., 2002; Link *et al*., 1999; Ceci *et al*., 2003; Shor *et al*., 2003; Baum *et al*., 2004; Gerbasi *et al*., 2004; Nilsson *et al*., 2004; Sengupta *et al*., 2004; Giavalisco *et al*., 2005; Yu *et al*., 2005). In this capacity, RACK1 might contribute to positional translation along the plasma membrane and perhaps within the mitotic apparatus. Recent studies indicate that ribosomes and specific mRNA transcripts associate with mitotic microtubules (Suprenant, 1993; Blower *et al*., 2007). When we began the current study, it was not possible to postulate a role for TbRACK1 in translational control as cryo-EM images of the trypanosome ribosome failed to detect it (Gao *et al*., 2005).

In an effort to understand the mechanisms by which TbRACK1 and its homologues might regulate cytokinesis, binding partners were sought. Mammalian RACK1 is multifunctional, with more than 25 known binding partners. This remarkable promiscuity allows RACK1 to contribute towards the regulation of a wide range of cell activities, including cell morphology, growth and gene expression. In trypanosomes the multifunctional properties of TbRACK1 are less clear. In the current report, we show that TbRACK1 is detected in sucrose gradient fractions that contain monosomes and polysomes; consistent with a role in translation. Additionally, TbRACK1 is found in non-ribosomal fractions; suggestive of roles beyond translation. We previously reported that TbRACK1 could complement CPC2/RACK1 null mutants of *S. pombe* and restore growth on minimal medium (Rothberg *et al*., 2006). These results indicate that TbRACK1 can perform the functions of Cpc2 in the context of *S. pombe*.

To identify the TbRACK1 target protein(s), we used a constitutively expressed TAP-TbRACK1. Tandem MS of the affinity eluate identified eEF1A as a consistent and predominant binding partner. The association between eEF1A and TbRACK1 was confirmed by the following experiments: co-sedimentation in sucrose gradients, co-precipitation in cell homogenates and coimmunoprecipitation of purified proteins. Other proteins identified in the TAP-TbRACK1 preparation included α- and β-tubulins, and these were not pursued further in this study. In neurons and yeast, mRNA-binding proteins formed complexes that also included RACK1, β-tubulin and eEF1A (Angenstein *et al*., 2002; Baum *et al*., 2004), while in HeLa cells, an E3 ligase had similar binding partners (Aranda-Orgillés *et al*., 2008). These studies did not determine whether RACK1 and eEF1A were in the same complex. The yeast two-hybrid databases for *S. cerevisiae*(http://www.yeastgenome.org) or *C. elegans*(http://www.wormbase.org) do not identify a direct interaction between RACK1 and eEF1A. However, both eEF1A and RACK1 can be post-translationally modified at multiple sites (Chang *et al*., 1998; Lamberti *et al*., 2004; Liu *et al*., 2007; Signorell *et al*., 2008). Therefore, the recombinant proteins from yeast and bacteria may not be suitable ligands for binding assays. We show that recombinant bacterially expressed eEF1A does not bind to TbRACK1 by bacterial two-hybrid or by immunoprecipitaton assays. The present report is the first to demonstrate that purified native eEF1A associates with RACK1.

The identification of eEF1A as a TbRACK1-associated protein is intriguing. RACK1 and eEF1A are each multifunctional proteins with activities that extend beyond their roles in translation. eEF1A has been reported to bundle actin filaments, sever microtubules, activate PI4-kinase and contribute towards various disease states (Reviewed in Ejiri, 2002; Lamberti *et al*., 2004). In trypanosomes, eEF1A has also been shown to be essential for the import of some tRNAs into the mitochondrion (Bouzaidi-Tiali *et al*., 2007). We previously reported that eEF1A from *T. brucei* associates with calmodulin (Kaur and Ruben, 1994) and this has been verified in plants (Durso and Cyr, 1994) and *Tetrahymena* (Numata *et al*., 2000). In *Tetrahymena*, eEF1A appears to be important for contractile ring formation during cytokinesis. Calmodulin interferes with the actin bundling properties of eEF1A (Kurasawa *et al*., 1996). In yeast, eEF1A appears to co-ordinate translation with integrity of the actin cytoskeleton (Gross and Kinzy, 2007). By contrast, the situation in trypanosomes is much less clear, as knockdown of the actin gene by RNAi does not produce a cytokinesis defect nor does it inhibit growth of PF trypanosomes (García-Salcedo *et al*., 2004). Instead actin is important for vesicle trafficking in BF trypanosomes. Therefore, the presence of an actin ring during cytokinesis has yet to be established in these organisms. The interaction between TbRACK1 and eEF1A might help co-ordinate translation and cytoskeletal events; however, this would depend upon whether TbRACK1 associates with ribosomes. We therefore re-evaluated the relationship between TbRACK1 and the protein translation machinery.

### TbRACK1 is part of the translation machinery in *T. brucei*

Trypanosomes diverged early in eukaryotic cell evolution. As a consequence they appear to have many unusual features; even in otherwise conserved pathways. While the overall process of gene expression is conserved in trypanosomes, it is distinguished by a number of novel components. The limited number of promoter regions minimizes the significance of transcriptional control mechanisms in trypanosomes, and requires the excision of individual transcripts from large polycistronic precursors. Each mature mRNA then results from addition of the same spliced leader RNA (SL RNA) to the 5′ UTR. The SL RNA receives the 7-methylguanosine (m7G) cap as in other higher eukaryotes, but then it is extensively modified on the first four nucleotides to form the novel cap 4 structure (Perry *et al*., 1987). Proteins that synthesize the cap 4 structure and those that bind to it also have novel components (Campbell *et al*., 2003; Li and Tschudi, 2005; Ruan *et al*., 2007). Mutated SL RNA and reduced cap 4 prevent the interactions between transcripts and polysomes, although a partially formed cap structure with wild-type SL RNA is sufficient for polysome binding (Zamudio *et al*., 2006).

The trypanosome ribosome has a generally conserved structure, but has a significantly larger volume than its yeast counterpart (Gao *et al*., 2005). Much of the volume change results from packing of the trypanosome rRNA expansion segments and their associated proteins. Unlike other ribosomes, the 25S rRNA precursor is processed into six structural rRNA species that range in size from 77 nucleotides to 1900 nucleotides in length. A trypanosome-specific phosphosphoprotein, called NOPP44/46 is required for this processing step (Jensen *et al*., 2005), while trypanosome-specific RNA-binding proteins regulate abundance of the 5S rRNA (Hellman *et al*., 2007). Noteworthy among the unusual structural features of the trypanosome ribosome is the absence of RACK1. Cryo-EM, tandem MS and Western blot had previously been used to detect RACK1 on ribosomes from yeast, plants and humans (Link *et al*., 1999: Giavalisco *et al*., 2005; Yu *et al*., 2005). Its absence from trypanosome ribosomes sets them apart from their counterparts in other taxa, and eliminates from consideration an important regulatory mechanism for translational control. In the present study, we report that TbRACK1 is a component of trypanosome ribosomes. In support of this contention, we demonstrate that: (i) TbRACK1 is found in a complex with the eukaryotic translation elongation factor-1a (eEF1A); (ii) sucrose gradient purified monosomes contain TbRACK1, which comigrates with the ribosomal marker TcP0 under conditions where cytoskeletal contaminants are not observed; (iii) TbRACK1 also comigrates with polysomes but not with cytoskeletal contaminants, and shifts distribution when polysomes are disrupted with RNase; (iv) when TbRACK1 is depleted with RNAi, the polysome pattern is consistent with a disruption of translation initiation; (v) the phosphorylation status of a 30 kDa ribosomal protein is dependent upon TbRACK1; and (vi) growth of the TbRACK1-depleted cells is hypersensitive to the translational inhibitor anisomycin;

Species variability cannot explain the difference between our results with *T. brucei* and those obtained by cryo-EM with *T. cruzi*. We also observe RACK1 on polysome preparations from *T. cruzi* epimastigotes. Methodology may account for some differences between the two studies. When our monosome preparations were treated with 0.2% of deoxycholate, similar to the lysis conditions with *T. cruzi*, the 80S ribosomes appear to lose TbRACK1. Alternatively, TbRACK1 may have a novel location on the ribosome, and therefore was not identified in the previous study.

### A link between translation and cytokinesis

The presence of TbRACK1 on trypanosome ribosomes indicates a mechanism for translational control of gene expression. This may be especially important in an organism where differential gene expression appears to be regulated by post-transcriptional processes. In yeast and mammals, the molecular steps by which RACK1 regulates translation is not clear, although knockout of its gene changes the expression pattern of several proteins (Shor *et al*., 2003; Gerbasi *et al*., 2004). The ability of RACK1 to bind ribonucleoprotein complexes may contribute towards translational control (Angenstein *et al*., 2002; Baum *et al*., 2004). In yeast and mammalian cells, RACK1 also plays a variety of roles in signal processes that appear to be unrelated to ribosome function (Koehler and Moran, 2001; Patterson *et al*., 2004; Chen *et al*., 2005; Bolger *et al*., 2006; Hu *et al*., 2006; Zeller *et al*., 2007). Our initial studies on TbRACK1 did not hint at a function in translation. We previously reported that the conditional knockdown of TbRACK1 with RNAi, caused approximately 15–20% of cells to arrest during cytokinesis, partway through the process of cleavage furrow ingression (Rothberg *et al*., 2006). These cells reentered the cell cycle, continued to divide their nuclei, assembled new flagella and arrested again during cytokinesis. The low percentage of cells that exhibited this phenotype may have resulted from a threshold phenomenon in which residual TbRACK1 was sufficient to maintain cytokinesis in most cells. In the present study, we report that the addition of subtoxic concentrations of the translational inhibitor anisomycin augments the cytokinesis defect observed with TbRACK1 RNAi. Under these conditions, approximately 60% of cells become multinucleate and multiflagellate. The effect of anisomycin appears to be specific as the cells are metabolically active (fully motile), and nuclear replication and flagellar assembly continue unabated. Moreover, anisomycin does not merely lower the levels of TbRACK1 in the RNAi cells. Therefore, anisomycin and TbRACK1 RNAi behave synergistically to regulate the last stage of cell division. We propose that TbRACK1 is required for efficient translation of specific transcripts, at least one of which is required for the latter stages of cytokinesis. In this model, RNAi of TbRACK1 lowers the expression level of this protein close to a threshold amount. The further addition of subtoxic levels of anisomycin in combination with RNAi of TbRACK1 lowers synthesis of the specific cell division protein below the threshold concentration, resulting in a cytokinesis defect. The expression level of the cytokinesis protein may be directly controlled by translation, or the protein may be rapidly degraded and depend upon continued translation to maintain a steady-state level. In organisms ranging from yeast to humans, translational control plays a key role in cell proliferation. The eIF4 initiation factors have been shown to play an especially important role in cell cycle control (Flynn and Proud, 1996; Lee and McCormick, 2006; Graff *et al*., 2008). In trypanosomatids, conserved components of the eIF4 complex have been identified and characterized (Dhalia *et al*., 2005; 2006).

Identification of cytokinesis-specific proteins is complicated in *T. brucei* by the ease with which this phenotype arises when a wide range of unrelated proteins are genetically knocked down (reviewed in Hammarton *et al*., 2007). The situation results from the dependence of cytokinesis upon disparate cell activities, including replication and segregation of single-copy organelles. While knockdown of many proteins by RNAi can generate ‘monster cells’ that are multinucleate, multiflagellate and misshapen, the effects on cytokinesis are often indirect. In the present study, subtoxic levels of anisomycin triggered a cytokinesis defect in cells that had previously been depleted of TbRACK1. The rapid onset of this defect allowed us to look at early times in the division arrest process. We noted that within 12 h of anisomycin treatment, only a small percentage of cells were in a precytokinesis 2N2K configuration, suggesting that they traversed this phase of the cell cycle without difficulty. Among these cells, 9% had an unusual KKNN configuration in which one of the daughter nuclei failed to migrate between the newly separated kinetoplasts. Because the two nuclei were not positioned properly relative to the kinetoplasts, the cleavage plane may not have aligned properly. Consequently, cleavage ingression stalled at the paired nuclei in 68% of all 2N2K cells, and stalled just beyond the nuclei in 18% of all 2N2K cells. Genetic disruption of MOB1, Tb-dynamin-like protein, or the initial step in sphingolipid biosynthesis generates a phenotype similar to the one we report for the knockdown of TbRACK1 (Hammarton *et al*., 2005; Chanez *et al*., 2006; Fridberg *et al*., 2008). It is not known whether any of these proteins either bind with TbRACK1 or have their synthesis regulated by TbRACK1. In yeast, it is of interest that the TOR signalling pathway contributes towards translational control of cell cycle progression and towards ceramide biosynthesis (Aronova *et al*., 2008; Jacinto and Lorberg, 2008).

In conclusion, we have shown that TbRACK1 is a component of the translation machinery in *T. brucei*. It forms a complex with eEF1A, and associates with monosomes or polysomes. The association with ribosomes appears to be important for their function. In the absence of TbRACK1, the initiation of translation and phosphorylation of a ribosomal protein are each disrupted. TbRACK1 also contributes towards the process of cytokinesis. Its conditional knockdown by RNAi generates a distinct phenotype characterized by partial ingression of the cleavage furrow. The translational inhibitor anisomycin augments the cytokinesis defect. Prior to cytokinesis, a postmitotic nucleus fails to migrate between the kinetoplasts. The misalignment of nuclei and kinetoplasts suggests a mechanism by which complete cleavage furrow ingression is prevented. Altogether, this is the first report to demonstrate that TbRACK1 is a component of trypanosome ribosomes and to demonstrate that continued translation is required to traverse a specific stage in the trypanosome cell cycle.

## Experimental procedures

### Cells and culture conditions

Cells used in this study include *T. brucei* PF derived from AnTat 1.1 BF (kindly provided by E. Pays, Free University of Brussels); engineered 29-13 PF that express the T7 RNA polymerase and tetracycline repressor protein (kindly provided by G.A.M. Cross, The Rockefeller University); monomorphic BF M110; and *T. cruzi* epimastigotes Telahuan strain. Wild-type PF cells were maintained in SDM-79 medium at 28°C in an atmosphere of 6.5% CO_2_. When needed, hygromycin was added at 50 μg ml^−1^, G418 was at 15 μg ml^−1^ and phleomycin was at 2.5 μg ml^−1^. RNAi was induced with 1 μg ml^−1^ tetracycline. BF trypanosomes were obtained from rodent blood following DE-52 anion exchange chromatography, as described previously (Rothberg *et al*., 2006). *T. cruzi* epimastigotes were grown in LDNT medium (0.5% liver extract, 0.4% NaCl, 0.5% tryptone, 0.04% KCl, 2% Na_2_HPO_4_.12H_2_O, 0.1% Pen-Strep (1000× stock), hemin (0.154% v/v of a 13 mg ml^−1^ stock solution).

### DNA constructs and recombinant proteins

The RNAi of TbRACK1 was described previously (Rothberg *et al*., 2006). A three-times cloned cell line was generated for these studies. The TAP-TbRACK1 construct was prepared by PCR amplification from genomic DNA of the complete coding sequence for TbRACK1 (Tb11.01.3170). The PCR product was cloned into the PmeI/BamHI sites of pcDNA3-NTAP (kindly provided by Anne-Claude Gingras, Samuel Lunenfeld Research Institute). The complete sequence for TAP-TbRACK1 was PCR amplified and cloned into the EcoRV/BamHI sites of pTSA.HYG (Sommer *et al*., 1992). The forward primer was 5′-GCAT**GATATC**ATGAAAGCTGATGCGCAACA-3′ and reverse was 5′-GCTA**GGATCC**TCACGCGTTCTCCGATACACCCCAGAC3′. The restriction sites are shown in bold type. The BssHII linearized pTSA.TAP-TbRACK1 integrated into the tubulin intergenic region, and constitutive expression was driven by a PARP promoter. The construct was electroporated into cultured AnTat1.1 PF as described previously (Rothberg *et al*., 2006). To express AU1-eEF1A in PF cells, the full-length coding sequence corresponding to Tb10.70.5670 was PCR amplified from genomic DNA. The construct was cloned into the HindIII/BamHI sites of the tetracycline inducible expression vector pLEW100. The forward primer was 5′-GACA**AAGCTT**ATG*GACACGTACCGCTACATT*GGAAAGGAAAAGGTGCACATG-3′ and the reverse primer was 5′-TGTC**GGATCC**TTATTTCTTCGAAGCCTTCACCGCAGCC-3′. Restriction sites are shown in bold and the AU1-tag is shown in italics. A bacterial cell line expressing (His)_6_-TbRACK1 was kindly prepared by A. Joachimiak, Argonne National Laboratory as part of a high throughput protein crystallization program. Full-length TbRACK1 was cloned into pMCSG19 (Nallamsetty *et al*., 2004; Donnelly *et al*., 2006), to produce a fusion protein comprised of the following components: maltose binding protein-TVMV protease site-(His)_6_-tag-TEV protease site-TbRACK1. pMCSG19.TbRACK1 was transformed into *E. coli* BL21(DE3) which already expressed the TVMV protease on pRK1037. The TbRACK1 fusion protein was cleaved *in vivo* with TVMV, and was purified by immobilized metal-ion affinity chromatography (IMAC). To express recombinant (His)_6_-eEF1A, the full-length coding sequence was cloned into the BamHI/NdeI restriction endonuclease sites of pET-16b. Protein expression was induced with 0.2 mM IPTG for 18 h at 25°C. Protein was purified by IMAC. The interaction between recombinant TbRACK1 and eEF1A was evaluated with the BacterioMatch II Two-Hybrid System (Stratagene). TbRACK1 was cloned into the NotI/XhoI sites on bait plasmid pBT, while eEF1A was cloned into the NotI/XhoI sites of the prey plasmid pTRG. Each vector was separately cloned into XL1-Blue MR cells and selected on LB plates with 10 μg ml^−1^ chloramphenicol (pBT) or 10 μg ml^−1^ tetracycline (pTRG). The BactrioMatch II validation reporter competent cells (Stratagene) were co-transformed with bait and prey plasmids following the manufacturer's instructions.

### Purification of TAP-TbRACK1 and its binding partners

A 2 l culture of a three-times cloned cell line expressing TAP-TbRACK1 was grown to late log phase (approximately 2 × 10^10^ cells, total). The cell pellet was washed 2×, 50 ml each with Dulbecco's PBS containing 5.6 mM glucose (Invitrogen). The final cell pellet was suspended at a volume of 6 ml in lysis buffer [10 mM Tris, 150 mM NaCl, 2 mM EDTA, 1 mM DTT, 10 mM NaF, 0.25 mM Na orthovanadate, 5 nM okadaic acid and 1× protease inhibitor cocktail (Sigma P8340), pH 8.0]. Cell homogenates were lysed by sonication with four pulses of 10 s duration, centrifuged at 10 000 *g* for 10 min and the supernatant was centrifuged at 30 000 *g* for 20 min. The 30K supernatant was processed as described by Puig *et al*., 2001, with some modifications. The supernatant was divided into four aliquots and each was added to 100 μl of washed, packed IgG Sepharose 6 Fast Flow beads (Amersham) and gently mixed for 4 h at 4°C. The IgG beads were washed by centrifugation 10× with 1 ml each of lysis buffer (1500 r.p.m. for 2 min at 4°C) followed by five washes with 1 ml each TEV buffer (10 mM Tris, 150 mM NaCl, 0.5 mM EDTA and 1 mM DTT, pH 8.0). TAP-TbRACK1 and associated proteins were eluted overnight with 100 U of AcTEV protease (Invitrogen) at 4°C in 0.3 ml TEV buffer. The IgG beads were eluted 3×, 300 μl each in TEV buffer. The combined eluates were brought to 6.0 mM CaCl_2_ in 1.2 ml TEV buffer and each of four aliquots was incubated with 100 μl of calmodulin-Sepharose beads (Amersham) for 3 h at 4°C. The calmodulin beads were transferred to a 0.8 × 4 cm Poly Prep chromatography column (Bio-Rad) and washed 10×, 1 ml each with calmodulin-binding buffer (10 mM Tris, 150 mM NaCl, 10 mM β-mercaptoethanol, 1 mM Mg acetate, 1 mM imidazole, 2 mM CaCl_2_, pH 8.0), followed by five washes, 1 ml each with the same buffer containing 1 mM CaCl_2_, pH 8.0). For protein elution, the columns were sealed and incubated for 5 min with calmodulin elution buffer (10 mM Tris, 150 mM NaCl, 10 mM β-mercaptoethanol, 1 mM Mg acetate, 1 mM imidazole, 25 mM EGTA, pH 8.0). The column was eluted 5×, 200 μl each with calmodulin elution buffer. The combined EGTA eluates were concentrated to 100 μl with a Millipore BioMax filter (5000 MW cutoff), boiled in SDS sample buffer and separated on a 12% polyacrylamide gel.

### Identification of trypanosome proteins by LC MS/MS

Coomassie-stained proteins were excised from the gel and identified at the MS and Proteomics Resource Center of the W.M. Keck Foundation Biotechnology Resource Laboratory at Yale University. Briefly, the proteins were digested with trypsin in 10 mM ammonium bicarbonate, and 5 μl of the supernatant was directly injected onto a 100 μm × 150 mm Atlantis column (Waters) running at 500 nL min^−1^. Initial HPLC conditions were 95% buffer A and 5% buffer B with the following linear gradient: 3 min, 5% B; 43 min, 37% B; 75 min, 75% B; and 85 min, 95% B. Buffer A was 98% water, 2% acetonitrile, 0.1% acetic acid and 0.01% TFA. Buffer B was 80% acetonitrile, 20% water, 0.09% acetic acid and 0.01% TFA. Data-dependent acquisition was performed so that the mass spectrometer switched automatically from MS to MS/MS modes when the total ion current increased above the 1.5 counts per second threshold set point. In order to obtain good fragmentation, a collision energy ramp was set for the different mass sizes and charge states, giving preference to doubly and triply charged species for fragmentation over singly charged. All MS/MS data were searched in-house using the Mascot algorithm for un-interpreted MS/MS spectra after using the Mascot Distiller program to generate Mascot compatible files. The Mascot Distiller program combined sequential MS/MS scans from profile data that had the same precursor ion. A charge state of +2 and +3 was preferentially located with a signal to noise ratio of 1.2 or greater and a peak list was generated for database searching. Both the NCBInr and *T. brucei* genome databases were searched. The Mascot significance score match is based on a MOWSE score and relies on multiple matches to more than one peptide from the same protein. Typical parameters used for searching are partial methionine oxidation and acrylamide modified cysteine, a peptide tolerance of ±0.6 Da, MS/MS fragment tolerance of ±0.4 Da and peptide charges of +2 or +3.

### Purification of eEF1A from trypanosome homogenates

eEF1A was purified from 2 × 10^10^ BF strain M110, essentially as described by us previously (Kaur and Ruben, 1994), except that the homogenization buffer was changed to 10 mM HEPES, pH 7.0, 1 mM EDTA, 1 mM dithiothreitol (DTT) and 1× protease inhibitor cocktail (Sigma). The cells were homogenized by osmotic lysis followed by sonication. The homogenate was centrifuged at 100 000 *g* for 1 h and the supernatant was applied to a DE-52 anion exchange column pre-equilibrated with homogenization buffer. The column was washed with 1 column volume of the same buffer. The column run-through and the wash were pooled and the total Ca^2+^ content was adjusted to 3 mM. The sample was loaded onto a calmodulin-Sepharose column (3 ml) pre-equilibrated with 50 mM Tris-HCl pH 7.0, 150 mM NaCl containing 0.2 mM CaCl_2_. The column was washed until no protein was detected by the Bradford assay (Bio-Rad). The column was eluted with the same buffer containing 2 mM EGTA and 1 ml fractions were collected. Final protein content was measured by the Bradford assay and purity assessed by SDS-PAGE and colloidal Coomassie staining. The procedure yielded ∼200 μg of native eEF1A.

### Immunoprecipitation assays

Endogenous TbRACK1 was immunoprecipitated from whole-cell homogenates and the associated eEF1A was identified by Western blot. PF (3 × 10^9^) were suspended in 400 μl hypotonic buffer (1 mM HEPES, 1 mM EDTA, pH 7.5), and lysis was achieved by passage through a 27-gauge needle, as described by Bangs *et al*. (1993). The cells were brought to 500 μl total volume with isotonic buffer (final concentration 25 mM HEPES, 100 mM sucrose, 80 mM potassium acetate, 1 mM EDTA, pH 7.5. The cell lysate was centrifuged at 10 000 *g* for 10 min. To 100 μl of the 10K supernatant was added combinations of rabbit anti-TbRACK1 (90 μg of the purified IgG fraction); 75 μl of washed protein A beads; and isotonic buffer, to a total volume of 500 μl. The samples were incubated overnight at 4°C and the beads were washed 5× in RIPA [50 mM Tris-HCl, 150 mM NaCl, 1 mM EDTA, 0.8% Triton X-100, 0.8% sodium deoxycholate, 0.1% SDS and 20 μl 1× protease inhibitor cocktail (Sigma), pH 7.4]. The washed beads were suspended in RIPA and an equal volume of 2× SDS-PAGE sample buffer. Proteins in the pull-down assay were detected by Western blot as outlined below. AU1-eEF1A was induced with 1 μg ml^−1^ tetracycline for 3 days and homogenates prepared as described above. Precipitation was with anti-AU1 beads (Covance). For immunoprecipitation assays with purified proteins, 8 μg of recombinant (His)_6_-TbRACK1 was added to either 10 μg of purified endogenous eEF1A or 8 μg of recombinant (His)_6_-eEF1A. Antibodies were 10 μg of rabbit anti-TbRACK1, or 10 μg of mouse anti-eEF1A (Upstate Biotechnology). After a 2 h incubation at 4°C, the protein complexes were bound with 50 μl of protein A-Sepharose beads (Sigma) 2 h; 4°C and washed 5× in RIPA. The beads were suspended in 30 μl of RIPA and an equal amount of 2× SDS-PAGE sample buffer. Proteins were analysed by SDS-PAGE and Western blot.

### Bacterial two-hybrid

The BacterioMatch II Two-Hybrid System was used (Stratagene). The bait was expressed as a fusion protein with the λ repressor (λcI) (pBT), while the prey was expressed as a fusion protein with the N-terminal domain of the α-subunit of RNA polymerase (pTRG). When bait and prey proteins interact, they recruit RNA polymerase to the promoter and activate transcription of the *HIS3* reporter gene and also the *aadA* streptomycin resistance gene; each expressed on the same F′ episome. The BactrioMatch II validation reporter competent cells (Stratagene) were co-transformed with bait and prey, and then plated on non-selective screening media comprised of M9^+^-defined medium without added histidine (His dropout), and containing an antibiotic appropriate for each plasmid (25 μg ml^−1^ chloramphenicol for pBT and 12.5 μg ml^−1^ tetracycline for pTRG). Ten random colonies from the non-selective plates were analysed further. The colonies were suspended in 50 μl of the non-selective medium and grown for 3 h at 37°C. The OD_630_ was determined and all suspension cultures were diluted to match the lowest OD630 value. A 5 μl aliquot of each culture was spotted onto selection plates comprised of either M9 ± His dropout supplemented with 5 mM 3-amino-1,2,4-triazole (3-AT); or M9 ± His dropout supplemented with 5 mM 3-AT plus 12.5 μg ml^−1^ streptomycin.

### Preparation of monosomes

Monosomes were prepared from trypanosome homogenates based upon published procedures (Brecht and Parsons, 1998; Rodríguez-Gabriel *et al*., 2000). Briefly, around 10^9^ log phase cells were incubated with 100 μg ml^−1^ cyclohexamide for 5 min, pelleted and washed once with polysome buffer (10 mM Tris-HCl, 300 mM KCl; 10 mM MgCl_2_, pH 7.4) supplemented with 100 μg ml^−1^ cycloheximide. The cells were suspended in 500 μl of polysome buffer containing 100 μg ml^−1^ cycloheximide, 1 mM DTT and protease inhibitor cocktail (Sigma-P2714). The cell suspension was vigorously mixed for 3 min with 425–600 μm glass beads (Sigma). Final lysis was achieved with 0.8% (v/v) Triton X-100 (Sigma). The cell lysate was centrifuged at 1000 *g* for 10 min and the supernatant was centrifuged twice at 14 000 *g* for 10 min. The final supernatant was brought to 1.5 ml in polysome buffer and centrifuged at 335 000 *g* (avg) for 30 min in the TL100.3 rotor (Beckman). The pellet was suspended in 500 μl of polysome buffer, and applied to a step gradient of sucrose (20% and 40%). After centrifugation at 335 000 *g* (avg) for 2 h, the pellet beneath the 40% layer was suspended in 500 μl of polysome buffer and applied a linear sucrose gradient (5–25%) prepared in polysome buffer plus 1× protease inhibitor cocktail (Sigma). The sucrose gradient was centrifuged for 2.5 h at 170 000 *g* (avg) in the SW41 rotor (Beckman). To disrupt the association between TbRACK1 and ribosomes, the ribosomes were treated with 0.7 M NaCl for 30 min, or with 0.25% (v/v) sodium deoxycholate for 45 min prior to centrifugation on the linear sucrose gradient. The gradients were harvested from the bottom through a capillary pipette with a peristaltic pump (ISCO). The OD_254_ was continuously recorded with a Knauer variable wavelength monitor attached to a Kipp and Zones BD40 chart recorder and 500 μl fractions were collected. The orientation of each chromatogram was flipped horizontally so that the top of the gradient is displayed on the left.

### Preparation of polysomes

The initial steps of the polysome preparations were identical to those for monosome preparations. After the second 14 000 *g* centrifugation step, 500 μl of the supernatant was applied to a 12 ml linear sucrose gradient (15–50%) prepared in polysome buffer and supplemented with protease inhibitor cocktail and RNAsin. The sucrose gradient was centrifuged for 2.5 h at 170 000 *g* (avg) in the SW41 rotor. The gradient was harvested as described for monosomes. In experiments where the polysomes were disrupted with RNase, 0.5 mg ml^−1^ RNase A was added for 30 min prior to centrifugation on the sucrose gradient as described (Shor *et al*., 2003).

### Gel electrophoresis, western blots and densitometry

Proteins in each sucrose fraction were concentrated by a methanol/chloroform precipitation method. Briefly, to each 500 μl sucrose gradient fraction was added 500 μl of methanol, followed by 100 μl of water-saturated chloroform, and then 400 μl of water. The samples were mixed vigorously after each addition. Protein was collected by centrifugation for 5 min at 10 000 *g*. The aqueous layer was discarded, and 500 μl of methanol was added to the protein disc and remaining organic layer. Following centrifugation for 5 min at 10 000 *g*, the pellet was allowed to air dry before it was mixed with 2× SDS-PAGE loading buffer and boiled for 10 min. Proteins were separated on 12% polyacrylamide gels and electrophoretically transferred to nitrocellulose membranes using the Bio-Rad semidry transfer apparatus for 20 min at 20 V. Blots were developed using the luminol/enhancer system (SuperSignal West Pico Chemilumnescence Kit, Pierce), following the manufacturer's instructions. Densitometry was performed on the X-ray film using the SpotDenso function of the AlphaImager 2200 (Alpha Innotech). An IDV value for each protein was determined and the amount of TbRACK1 in a sample was defined as the ratio of IDV values for TbRACK1:TcP0. Antibodies used in this study include rabbit anti-TbRACK1 (1:2000, Rothberg *et al*., 2006); mouse anti-EF1A (1: 2000, Upstate Biotechnology); rabbit anti-TcP0 (1:2000, kindly provided by S. Reed, Infectious Disease Research Institute); mouse anti-α-tubulin (1:2000; Sigma); mouse anti-phosphothreonine (1:1000, Sigma); rat anti-paraflagellar rod (kindly provided by T. Seebeck, University of Bern, 1:200); Secondary antibodies were purchased conjugated to horseradish peroxidase, Cy2 or Cy3 (Jackson Immunoabs).

### Analysis of TbRACK1 RNAi cells

For RNAi induction, cells were diluted to 5.5 × 10^5^ cells ml^−1^ and cultured in the presence of 1 μg ml^−1^ tetracycline (+Tet) or were untreated (−Tet). Cells were allowed to grow for 4 days to deplete the cellular content of TbRACK1. Polysomes were isolated from 1 × 10^9^ cells (+/−)Tet as described above. Monosomes from 1 × 10^9^ cells (+/−)Tet were collected after centrifugation through the sucrose cushion. To measure the effects of anisomysin treatment, log phase TbRACK1 RNAi cells were diluted 1:10 and grown overnight. The overnight culture was used to seed two 5 ml cultures, each at a density of 1 × 10^6^ cells ml^−1^. Tetracylcine (1 μg ml^−1^) was added to one culture. After 2 days' growth, the cultures were again diluted to 1 × 10^6^ cells ml^−1^ and seeded onto a 24-well plate. Duplicate wells received 0, 4, 6, 12 or 20 μg ml^−1^ anisomycin. After an additional 48 h growth, the cells were counted. Cells were also fixed for microscopy as described below.

### Microscopy

Cells were washed with phosphate buffered saline (PBS with Dulbecco's salts; Invitrogen) and fixed for 45 min with 4% paraformaldehyde in the same buffer. After washing the cells in 50 mM Tris-HCl, 150 mM NaCl, pH 7.5, the cells were allowed to settle for 1 h on Fisher (+) Gold positively charged microscope slides. The fixed cells were permeabilized on the slide with 0.1% Igepal CA-630 (Sigma) in PBS and blocked with 4% goat serum in PBS. Primary antibody was added in the presence of 0.2% gelatin for 1 h at 37°C. After three washes in PBS plus gelatin, cells were treated with secondary antibodies (Cy2 or Cy3, Jackson Immunoabs), counterstained with the DNA-specific dye TOTO (Molecular Probes), and washed three more times. Cells were coated with Mounting Medium (Kirkegaard and Perry Laboratories, MD) prior to viewing. Microscopy was with a Nikon C1 Digital Eclipse Confocal E600 microscope. Images were collected with EZ-C1 software (Nikon).

## References

[b1] Akopyants NS, Matlib RS, Brownstein BH, Stormo GD, Beverley SM (2004). Expression profiling using a random genomic DNA microarray identifies differentially expressed genes associated with three major developmental stages of the protozoan parasite *Leishmania major*. Mol Biochem Parasitol.

[b2] Angenstein F, Evans AM, Settlage RE, Moran ST, Ling SC, Klintsova AY (2002). A receptor for activated C kinase is part of messenger ribonucleoprotein complexes associated with polyA-mRNAs in neurons. J Neurosci.

[b3] Aranda-Orgillés B, Trockenbacher A, Winter J, Aigner J, Kohler AL, Jastrzebska E (2008). The Opitz syndrome gene product MID1 assembles a microtubule-associated ribonucleoprotein complex. Hum Genet.

[b4] Aronova S, Wedaman K, Aronov PA, Fontes K, Ramos K, Hammock BD, Powers T (2008). Regulation of ceramide biosynthesis by TOR complex 2. Cell Metab.

[b5] Avila AR, Yamada-Ogatta SF, Monteiro V, Krieger MA, Nakamura CV, de Souza W, Goldenberg S (2001). Cloning and characterization of the metacyclogenin gene, which is specifically expressed during *Trypanosoma cruzi* metacyclogenesis. Mol Biochem Parasitol.

[b6] Ayub MJ, Smulski CR, Nyambega B, Bercovich N, Masiga D, Vazquez MP (2005). Protein–protein interaction map of the *Trypanosoma cruzi* ribosomal P protein complex. Gene.

[b7] Bangs JD, Uyetake L, Brickman MJ, Balber AE, Boothroyd JC (1993). Molecular cloning and cellular localization of a BiP homologue in *Trypanosoma brucei*. Divergent ER retention signals in a lower eukaryote. J Cell Sci.

[b8] Baum S, Bittins M, Frey S, Seedorf M (2004). Asc1p, a WD40-domain containing adaptor protein, is required for the interaction of the RNA-binding protein Scp160p with polysomes. Biochem J.

[b9] Blower MD, Feric E, Weis K, Heald R (2007). Genome-wide analysis demonstrates conserved localization of messenger RNAs to mitotic microtubules. J Cell Biol.

[b10] Bolger GB, Baillie GS, Li X, Lynch MJ, Herzyk P, Mohamed A (2006). Scanning peptide array analyses identify overlapping binding sites for the signalling scaffold proteins, beta-arrestin and RACK1, in cAMP-specific phosphodiesterase PDE4D5. Biochem J.

[b11] Bouzaidi-Tiali N, Aeby E, Charriere F, Pusnik M, Schneider A (2007). Elongation factor 1a mediates the specificity of mitochondrial tRNA import in *T. brucei*. EMBO J.

[b12] Brecht M, Parsons M (1998). Changes in polysome profiles accompany trypanosome development. Mol Biochem Parasitol.

[b13] Brems S, Guilbridge DL, Gundlesdodjir-Planck D, Busold C, Luu VD, Schanne M (2005). The transcriptomes of *Trypanosoma brucei* Lister 427 and TREU927 bloodstream and procyclic trypomastigotes. Mol Biochem Parasitol.

[b14] Campbell DA, Thomas S, Sturm NR (2003). Transcription in the kinetoplastid protozoa: why be normal?. Microbes Infect.

[b15] Ceci M, Gaviraghi C, Gorrini C, Sala LA, Offenhauser N, Marchisio PC, Biffo S (2003). Release of eIF6 (p27BBP) from the 60S subunit allows 80S ribosome assembly. Nature.

[b16] Chanez AL, Hehl AB, Engstler M, Schneider A (2006). Ablation of the single dynamin of *T. brucei* blocks mitochondrial fission and endocytosis and leads to a precise cytokinesis arrest. J Cell Sci.

[b17] Chang BY, Conroy KB, Machleder EM, Cartwright CA (1998). RACK1, a receptor for activated C kinase and a homolog of the β-subunit of G proteins, inhibits activity of *src* tyrosine kinases and growth of NIH 3T3 cells. Mol Cell Biol.

[b18] Chantrel Y, Gaisne M, Lions C, Verdiere J (1998). The transcriptional regulator Hap1p (Cyp1p) is essential for anaerobic or heme-deficient growth of *Saccharomyces cerevisiae*: genetic and molecular characterization of an extragenic suppressor that encodes a WD repeat protein. Genetics.

[b19] Chen S, Lin F, Hamm HE (2005). RACK1 binds to a signal transfer region of Gβγ and inhibits phospholipase C β2 activation. J Biol Chem.

[b20] Chicurel ME, Singer RH, Meyer CJ, Ingber DE (1998). Integrin binding and mechanical tension induce movement of mRNA and ribosomes to focal adhesions. Nature.

[b21] Clayton CE (2002). Life without transcriptional control? From fly to man and back again. EMBO J.

[b22] Clayton C, Shapira M (2007). Post-transcriptional regulation of gene expression in trypanosomes and leishmanias. Mol Biochem Parasitol.

[b23] Cohen-Freue G, Holzer TR, Forney JD, McMaster WR (2007). Global gene expression in *Leishmania*. Int J Parasitol.

[b24] Cox EA, Bennin D, Doan AT, O'Toole T, Huttenlocher A (2003). RACK1 regulates integrin-mediated adhesion, protrusion and chemotactic cell migration via its Src-binding site. Mol Biol Cell.

[b25] Dhalia R, Marinsek N, Reis CRS, Katz R, Muniz JRC, Standart N (2006). The two eIF4A helicases in *Trypanosoma brucei* are functionally distinct. Nucleic Acids Res.

[b26] Dhalia R, Reis CR, Freire ER, Rocha PO, Katz R, Muniz JR (2005). Translation initiation in *Leishmania major*: characterization of multiple eIF4F subunit homologues. Mol Biochem Parasitol.

[b27] Donnelly MI, Zhou M, Millard CS, Clancy S, Stols L, Eschenfeldt WH (2006). An expression vector tailored for large-scale high-throughput purification of recombinant proteins. Protein Expr Purif.

[b28] Dresios J, Panopoulos P, Synetos D (2006). Eukaryotic ribosomal proteins lacking a eubacterial counterpart: important players in ribosomal function. Mol Microbiol.

[b29] Durso NA, Cyr RJ (1994). A calmodulin-sensitive interaction between microtubules and a higher plant homolog of elongation factor-1α. Plant Cell.

[b30] Ejiri S (2002). Moonlighting functions of polypeptide elongation factor 1: from actin bundling to zinc finger protein R1-associated nuclear localization. Biosci Biotechnol Biochem.

[b31] Flynn A, Proud CG (1996). The role of eIF4 in cell proliferation. Cancer Surv.

[b32] Fomenkov A, Zangen R, Huang YP, Osada M, Guo Z, Fomenkov T (2004). RACK1 and stratifin target ΔNp63α for proteasome degradation in head and neck squamous cell carcinoma cells upon DNA damage. Cell Cycle.

[b33] Fridberg A, Olson CL, Nakayasu ES, Tyler KM, Almeida IC, Engman DM (2008). Sphingolipid synthesis is necessary for kinetoplast segregation and cytokinesis in *Trypanosoma brucei*. J Cell Sci.

[b34] Gale M, Carter V, Parsons M (1994). Translational control mediates the developmental regulation of *Trypanosoma brucei* Nrk protein kinase. J Biol Chem.

[b35] Gao H, Ayub MJ, Levin MJ, Frank J (2005). Structure of the 80S ribosome from *Trypanosoma cruzi* reveals novel rRNA components involved in translation initiation. Proc Natl Acad Sci USA.

[b36] García-Salcedo JA, Pérez-Morga D, Gijón P, Dilbeck V, Pays E, Nolan DP (2004). A differential role for actin during the life cycle of *Trypanosoma brucei*. EMBO J.

[b37] Gavin AC, Aloy P, Grandi P, Krause R, Boesch M, Marzioch M (2006). Proteome survey reveals modularity of the yeast cell machinery. Nature.

[b38] Gerbasi VR, Weaver CM, Hill S, Friedman DB, Link AJ (2004). Yeast Asc1p and mammalian RACK1 are functionally orthologous core 40S ribosomal proteins that repress gene expression. Mol Cell Biol.

[b39] Giavalisco P, Wilson D, Kreitler T, Lehrach H, Klose J, Gobom J, Fucini P (2005). High heterogeneity within the ribosomal proteins of the *Arabidopsis thaliana* 80S ribosome. Plant Mol Biol.

[b40] Graff JR, Konicek BW, Carter JH, Marcusson EG (2008). Targeting the eukaryotic translation initiation factor 4E for cancer therapy. Cancer Res.

[b41] Gross SR, Kinzy TG (2007). Improper organization of the actin cytoskeleton affects protein synthesis at initiation. Mol Cell Biol.

[b42] Hammarton TC, Lillico SG, Welburn SC, Mottram JC (2005). Trypanosoma brucei MOB1 is required for accurate and efficient cytokinesis but not for exit from mitosis. Mol Microbiol.

[b43] Hammarton TC, Monnerat S, Mottram JC (2007). Cytokinesis in trypanosomatids. Curr Opin Microbiol.

[b44] Hellman KM, Ciganda M, brown SV, Li J, Ruyechan W, Williams N (2007). Two trypanosome-specific proteins are essential factors for 5S rRNA abundance and ribosomal assembly in *T. brucei*. Eukaryot Cell.

[b45] Hu L, Lu F, Wang Y, Liu Y, Liu D, Jiang Z (2006). RACK1, a novel hPER1-interacting protein. J Mol Neurosci.

[b46] Inada T, Winstall E, Tarun SZ, Yates JR, Schieltz D, Sachs AB (2002). One-step affinity purification of the yeast ribosome and its associated proteins and mRNAs. RNA.

[b47] Isacson CK, Lu Q, Karas RH, Cox DH (2007). RACK1 is a BKCa channel binding protein. Am J Physiol Cell Physiol.

[b48] Jacinto E, Lorberg A (2008). TOR regulation of AGC kinases in yeast and mammals. Biochem J.

[b49] Jensen BC, Wang Q, Brekken DL, Randall AC, Kifer CT, Parsons M (2005). Species specificity in ribosome biogenesis: a nonconserved phosphoprotein is required for formation of the large ribosomal subunit in *Trypanosoma brucei*. Eukaryot Cell.

[b50] Kaur KJ, Ruben L (1994). Protein translation elongation factor-1 alpha from *Trypanosoma brucei* binds calmodulin. J Biol Chem.

[b51] Kelly BL, Stetson DB, Locksley RM (2003). *Leishmania major* LACK antigen is required for efficient vertebrate parasitization. J Exp Med.

[b52] Kiely PA, Baillie GS, Lynch MJ, Houslay MD, O'Connor R (2008). Tyrosine 302 in Rack1 is essential for IGF-I-mediated competitive binding of PP2A and beta 1 integrin and for tumour cell proliferation and migration. J Biol Chem.

[b53] Koehler J, Moran MF (2001). RACK1, a protein kinase C scaffolding protein, interacts with the PH domain of p120GAP. Biochem Biophys Res Commun.

[b54] Kurasawa Y, Hanyu K, Watanabe Y, Numata O (1996). F-actin bundling activity of *Tetrahymena* elongation factor 1 alpha is regulated by Ca^2+^/calmodulin. J Biochem.

[b55] Lamberti A, Caraglia M, Longo O, Marra M, Abbruzzese A, Aracari P (2004). The translation elongation factor 1A in tumorigenesis, signal transduction and apoptosis: review article. Amino Acids.

[b56] Lee SH, McCormick F (2006). p97/DAP5 is a ribosome-associated factor that facilitates protein synthesis and cell proliferation by modulating the synthesis of cell cycle proteins. EMBO J.

[b57] Li H, Tschudi C (2005). Novel and essential subunits in the 300-kilodalton nuclear cap binding complex of *Trypanosoma brucei*. Mol Cell Biol.

[b58] Liliental J, Chang DD (1998). Rack1, a receptor for activated protein kinase C, interacts with integrin-β subunit. J Biol Chem.

[b59] Link AJ, Eng J, Schieltz DM, Carmack E, Mize GJ, Morris DR (1999). Direct analysis of protein complexes using mass spectrometry. Nat Biotechnol.

[b60] Liu VY, Hubbi ME, Pan F, McDonald KR, Mansharamani M, Cole RN (2007). Calcineurin promotes hypoxia-inducible factor 1 alpha expression by dephosphorylating RACK1 and blocking RACK1 dimerization. J Biol Chem.

[b61] Loreni F, Iadevaia V, Tino E, Caldarola S, Amaldi F (2005). RACK1 mRNA translation is regulated via a rapamycin-sensitive pathway and coordinated with ribosomal protein synthesis. FEBS Lett.

[b62] Matthews KR, Gull K (1998). Identification of stage-regulated and differentiation-enriched transcripts during transformation of the African trypanosome from its bloodstream to procyclic form. Mol Biochem Parasitol.

[b63] Mayho M, Fenn K, Craddy P, Crosthwaite S, Matthews K (2006). Post-transcriptional control of nuclear-encoded cytochrome oxidase subunits in *Trypanosoma brucei*: evidence for genome-wide conservation of life-cycle stage-specificity regulatory elements. Nucleic Acids Res.

[b64] McLeod M, Shor B, Caporaso A, Wang W, Chen H, Hu L (2000). Cpc2, a fission yeast homologue of mammalian RACK1 protein, interacts with Ran1 (Pat1) kinase to regulate cell cycle progression and meiotic development. Mol Cell Biol.

[b65] McNicoll F, Drummelsmith J, Müller M, Madore E, Boilard N, Ouellette M, Papadopoulou B (2006). A combined proteomic and transcriptomic approach to the study of stage differentiation in *Leishmania infantum*. Proteomics.

[b66] Mourton T, Hellberg CB, Burden-Gulley SM, Himman J, Rhee A, Brady-Kalnay SM (2001). The PTPμ protein-tyrosine phosphatase binds and recruits the scaffolding protein RACK1 to cell-cell contacts. J Biol Chem.

[b67] Nallamsetty S, Kapust RB, Tözsér J, Cherry S, Tropea JE, Copeland TD, Waugh DS (2004). Efficient site-specific processing of fusion proteins by tobacco vein mottling virus protease *in vivo* and *in vitro*. Protein Expr Purif.

[b68] Naranda T, Kainuma M, MacMillan SE, Hershey JW (1997). The 39-kilodalton subunit of eukaryotic translation initiation factor 3 is essential for the complex's integrity and for cell viability in *Saccharomyces cerevisiae*. Mol Cell Biol.

[b69] Nardelli SC, Avila AR, Freund A, Motta MC, Manhaes L, de Jesus TC (2007). Small-subunit rRNA processome proteins are translationally regulated during differentiation of *Trypanosoma cruzi*. Eukaryot Cell.

[b70] Nelson RJ, Ziegelhoffer T, Nicolet C, Werner-Washburne M, Craig EA (1992). The translational machinery and 70 Kd heat shock protein cooperate in protein synthesis. Cell.

[b71] Nilsson J, Sengupta J, Frank J, Nissen P (2004). Regulation of eukaryotic translation by the RACK1 protein: a platform for signaling molecules on the ribosome. EMBO Rep.

[b72] Numata O, Kurasawa Y, Gonda K, Watanabe Y (2000). *Tetrahymena* elongation factor-1alpha is localized with calmodulin in the division furrow. J Biochem.

[b73] Okano K, Schnaper HW, Bomsztyk K, Hayashida T (2006). RACK1 binds to Smad3 to modulate transforming growth factor-β1-stimulated α2(I) collagen transcription in renal tubular epithelial cells. J Biol Chem.

[b74] Onishi I, Lin PJ, Diering GH, Williams WP, Numata M (2006). RACK1 associates with NHE5 in focal adhesions and positively regulates the transporter activity. Cell Signal.

[b75] Paterou A, Walrad P, Craddy P, Fenn K, Matthews K (2006). Identification and stage-specific association with the translational apparatus of *Tb*ZFP3, a CCCH protein that promotes trypanosome life-cycle development. J Biol Chem.

[b76] Patterson RL, van Rossum DB, Barrow RK, Snyder SH (2004). RACK1 Binds to inositol 1,4,5-trisphosphate receptor and mediates Ca^2+^ release. Proc Natl Acad Sci USA.

[b77] Perry KL, Watkins KP, Agabian N (1987). Trypanosome mRNAs have unusual ‘cap 4’ structures acquired by addition of a spliced leader. Proc Natl Acad Sci USA.

[b78] Puig O, Caspary F, Rigaut G, Rutz B, Bouvert E, Bragado-Nilsson E (2001). The tandem affinity purification (TAP) method: a general procedure of protein complex purification. Methods.

[b79] Rigaut G, Shevchenko A, Rutz B, Wilm M, Mann M, Séraphin B (1999). A generic protein purification method for protein complex characterization and proteome exploration. Nat Biotechnol.

[b80] Rodríguez-Gabriel MA, Remacha M, Ballestra JP (2000). The RNA interacting domain but not the protein interacting domain is highly conserved in ribosomal protein P0. J Biol Chem.

[b81] Ron D, Chen CH, Caldwell J, Jamieson L, Orr E, Mochly-Rosen D (1994). Cloning of an intracellular receptor for protein kinase C: a homolog of the β-subunit of G proteins. Proc Natl Acad Sci USA.

[b82] Rothberg KG, Burdette DL, Pfannstiel J, Jetton N, Singh R, Ruben L (2006). The RACK1 homologue from *Trypanosoma brucei* is required for the onset and progression of cytokinesis. J Biol Chem.

[b83] Ruan JP, Shen S, Ullu E, Tschudi C (2007). Evidence for a capping enzyme with specificity for the trypanosome spliced leader RNA. Mol Biochem Parasitol.

[b84] Saas J, Ziegelbauer K, von Haeseler A, Fast B, Boshart M (2000). A developmentally regulated aconitase related to iron-regulatory protein-1 is localized in the cytoplasm and in the mitochondrion of *Trypanosoma brucei*. J Biol Chem.

[b85] Sengupta J, Nilsson J, Gurskey R, Spahn CM, Nissen P, Frank J (2004). Identification of the versatile scaffold protein RACK1 on the eukaryotic ribosome by cryo-EM. Nat Struct Mol Biol.

[b86] Shor B, Calaycay J, Rushbrook J, McLeod M (2003). Cpc2/RACK1 is a ribosome-associated protein that promotes efficient translation in *Schizosaccharomyces pombe*. J Biol Chem.

[b87] Signorell A, Jennifer J, Rauch M, Bütikofer P (2008). Phosphatidylethanolamine in *Trypanosoma brucei* is organized in two separate pools and is synthesized exclusively by the Kennedy pathway. J Biol Chem.

[b88] Skeiky YA, Benson DR, Guderian JA, Sleath PR, Parsons M, Reed SG (1993). *Trypanosoma cruzi* acidic ribosomal P protein gene family. Novel P proteins encoding unusual cross-reactive epitopes. J Immunol.

[b89] Skop AR, Liu H, Yates J, Meyer BJ, Heald R (2004). Dissection of the mammalian midbody proteome reveals conserved cytokinesis mechanisms. Science.

[b90] Sommer JM, Cheng CL, Keller GA, Wang CC (1992). *In vivo* import of firefly luciferase into the glycosomes of *Trypanosoma brucei* and mutational analysis of the C-terminal targeting signal. Mol Biol Cell.

[b91] Spence J, Gali RR, Dittmar G, Sherman F, Karin M, Finley D (2000). Cell cycle-regulated modification of the ribosome by a variant multiubiquitin chain. Cell.

[b92] Stebbins EG, Mochly-Rosen D (2001). Binding specificity for RACK1 resides in the V5 region of beta II protein kinase C. J Biol Chem.

[b93] Suprenant KA (1993). Microtubules, ribosomes and RNA: evidence for cytoplasmic localization and translational regulation. Cell Motil Cytoskel.

[b94] Taladriz S, Gonzalez-Aseguinolaza G, Marquet A, Larraga V (1999). Cloning, molecular analysis and differential cell localisation of the p36 RACK analogue antigen from the parasite protozoon *Crithidia fasciculate*. FEBS Lett.

[b95] Valerius O, Kleinschmidt M, Rachfall N, Schulze F, López Marín S, Hoppert M (2007). The *Saccharomyces* homolog of mammalian RACK1, Cpc2/Asc1p, is required for FLO11 dependent adhesive growth and dimorphism. Mol Cell Proteomics.

[b96] Welburn SC, Murphy NB (1998). Prohibitin and RACK1 homologues are up-regulated in trypanosomes induced to undergo apoptosis and in naturally occurring terminally differentiated forms. Cell Death Differ.

[b97] Woodward R, Gull K (1990). Timing of nuclear and kinetoplast DNA replication and early morphological events in the cell cycle of *Trypanosoma brucei*. J Cell Sci.

[b98] Yaka R, Thornton C, Vagts AJ, Phamluong K, Bonci A, Ron D (2002). NMDA receptor function is regulated by the inhibitory scaffolding protein, RACK1. Proc Natl Acad Sci USA.

[b99] Yu Y, Ji H, Doudna JA, Leary JA (2005). Mass spectrometric analysis of the human 40S ribosomal subunit: native and HCV IRES-bound complexes. Protein Sci.

[b100] Zamudio JR, Mittra B, Zeiner GM, Geder M, Bujnicki JM, Sturm NR, Campbell DA (2006). Complete cap 4 formation is not required for viability in *Trypanosoma brucei*. Eukaryot Cell.

[b101] Zeller CE, Parnell SC, Dohlman HG (2007). The RACK1 ortholog Asc1 functions as a G-protein beta subunit coupled to glucose responsiveness in yeast. J Biol Chem.

[b102] Zhang W, Zong CS, Hermanto U, Lopez-Bergami P, Ronai Z, Wang LH (2006). RACK1 recruits STAT3 specifically to insulin and insulin-like growth factor 1 receptors for activation, which is important for regulating anchorage-independent growth. Molec Cell Biol.

