# Supplementary material for: The RACK1 signal anchor protein from Trypanosoma brucei associates with eukaryotic elongation factor 1A: a role for translational control in cytokinesis
Source: Mol Microbiol. 2008 Sep 25;70(3):724–45. doi: 10.1111/j.1365-2958.2008.06443.x (PMC2581647; doi:10.1111/j.1365-2958.2008.06443.x)
Supplement: Supplementary file 1 [file mmi0070-0724-SD1.pdf]

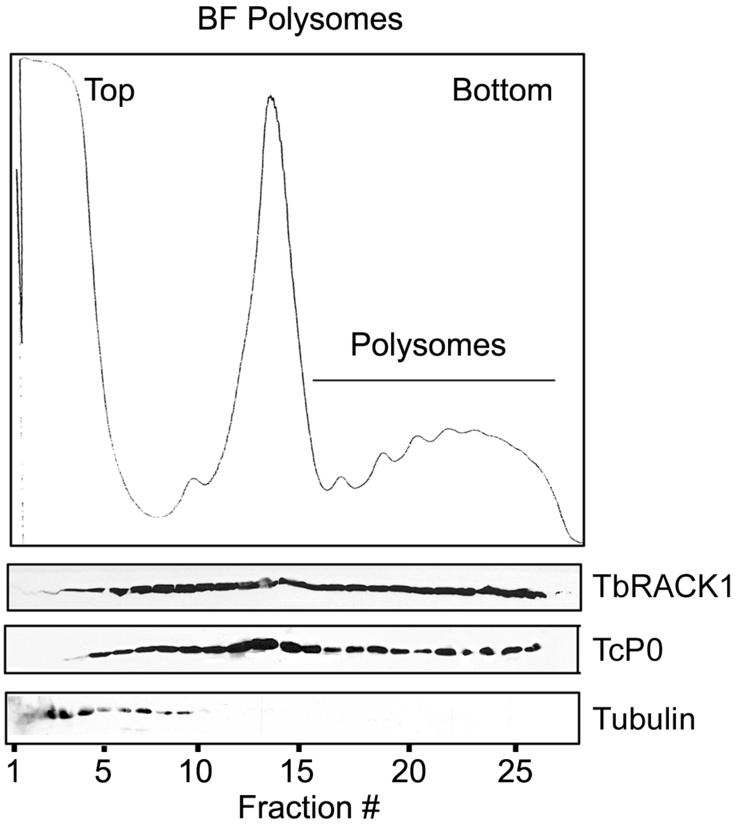


**Fig. S1.** Polysome preparations from BF contain TbRACK1.

Monomorphic BF strain M110 were harvested from rat blood and polysomes were prepared. The final 15-50% sucrose gradient is shown. The $OD_{254}$ was continuously recorded and 0.5 ml fractions were collected. Protein in each fraction was precipitated by chloroform/methanol and separated by SDS-PAGE. Western blot was used to detect TbRACK1, TcP0 or $\alpha$-tubulin in each fraction.
